# Supplementary material for: Osteopontin is a therapeutic target that drives breast cancer recurrence
Source: Nat Commun. 2024 Oct 24;15:9174. doi: 10.1038/s41467-024-53023-9 (PMC11502809; doi:10.1038/s41467-024-53023-9)
Supplement: Supplementary file 1 — Supplementary Information [file 41467_2024_53023_MOESM1_ESM.pdf]

Supplementary Information

Title: Osteopontin is a therapeutic target that drives breast cancer recurrence

Yu Gu [1, 2], Tarek Taifour [1, 3], Tung Bui [1, 2], Dongmei Zuo [1], Alain Pacis [1, 4], Alexandre Poirier [1, 3], Sherif Attalla [1], Anne-Marie Fortier [1], Virginie Sanguin-Gendreau [1], Tien-Chi Pan [5], Vasilios Papavasiliou [1], Nancy U Lin [6], Melissa E Hughes [6], Kalie Smith [6], Morag Park [1, 2, 7], Michel L. Tremblay [1, 2, 3, 7], Lewis A. Chodosh [5], Rinath Jeselsohn [6] and William J. Muller [1, 2, 7, *]

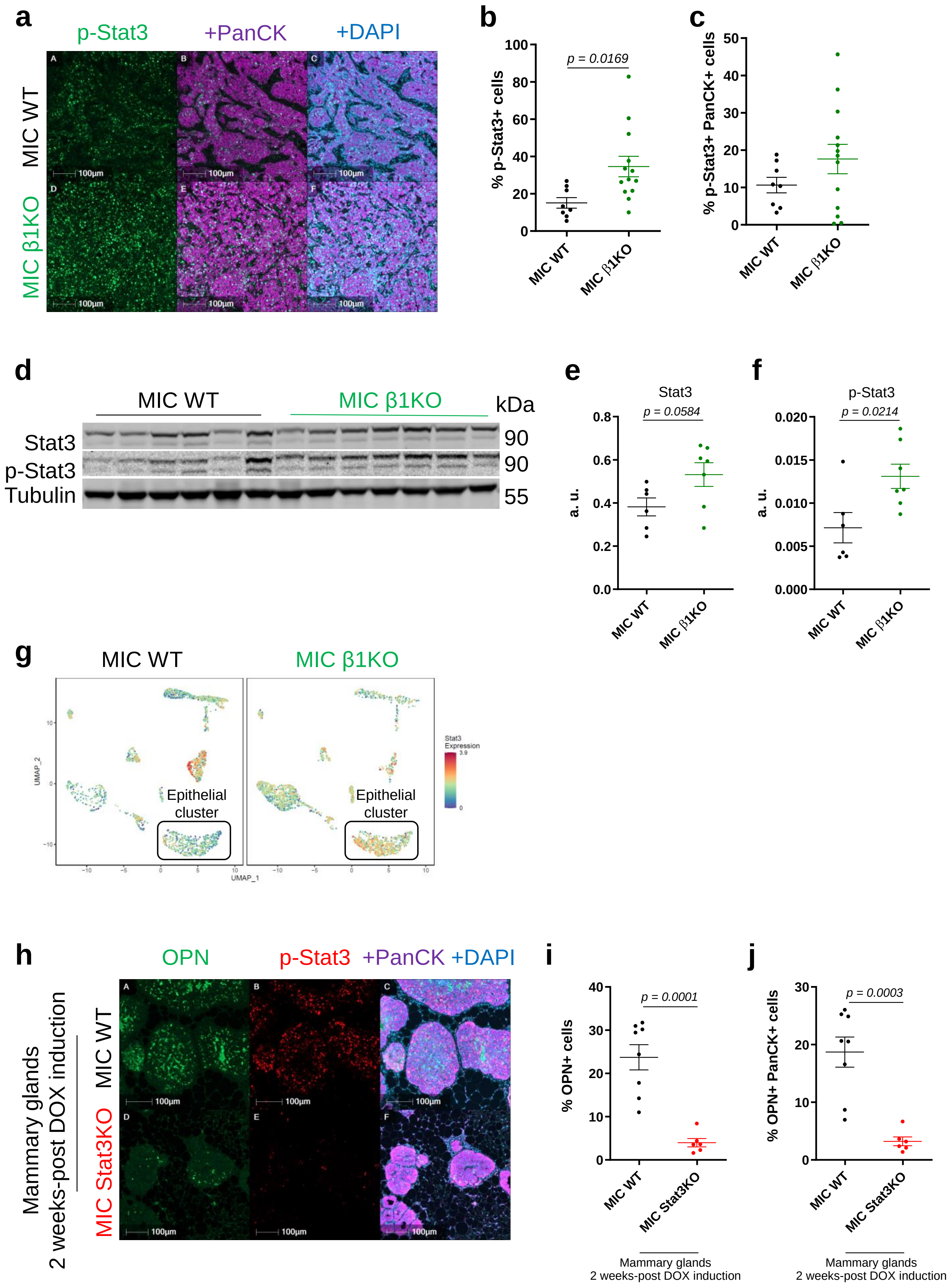
a
p-Stat3
+PanCK
+DAPI
MIC WT
MIC β1KO
A
B
C
D
E
F
100μm
b
% p-Stat3+ cells
p = 0.0169
100
80
60
40
20
0
MIC WT
MIC β1KO
c
% p-Stat3+ PanCK+ cells
50
40
30
20
10
0
MIC WT
MIC β1KO
d
MIC WT
MIC β1KO
kDa
Stat3
p-Stat3
Tubulin
90
90
55
e
Stat3
p = 0.0584
a. u.
0.8
0.6
0.4
0.2
0.0
MIC WT
MIC β1KO
f
p-Stat3
p = 0.0214
a. u.
0.020
0.015
0.010
0.005
0.000
MIC WT
MIC β1KO
g
MIC WT
MIC β1KO
UMAP_2
UMAP_1
Stat3 Expression
3.9
0
Epithelial cluster
Epithelial cluster
h
OPN
p-Stat3
+PanCK
+DAPI
Mammary glands 2 weeks-post DOX induction
MIC WT
MIC Stat3KO
A
B
C
D
E
F
100μm
i
% OPN+ cells
p = 0.0001
40
30
20
10
0
MIC WT
MIC Stat3KO
Mammary glands 2 weeks-post DOX induction
j
% OPN+ PanCK+ cells
p = 0.0003
30
20
10
0
MIC WT
MIC Stat3KO
Mammary glands 2 weeks-post DOX induction

Supplementary Figure 1. β1 integrin-deficient recurrent tumors have elevated levels of osteopontin transcription factor Stat3.

a Fluorescent immunohistochemistry (IHC) for p-Stat3, PanCK, and DAPI on MIC WT (n = 8) and MIC β1KO (n = 13) recurrent tumors. **b-c** Quantification of p-Stat3+ cells and p-Stat3+ PanCK+ cells in MIC WT (n = 8) and MIC β1KO (n = 13) recurrent tumors. **d** Immunoblot for Stat3 and p-Stat3 with loading control (tubulin) on MIC WT (n = 6) and MIC β1KO (n = 7) recurrent tumor lysates. **e-f** Quantification of immunoblot for Stat3 and p-Stat3 normalized to tubulin. **g** UMAP plots showing *Stat3* expression from single-cell RNA sequencing of early invasive carcinoma from MIC WT lesions (fast-growing, pooled lesions from n = 3 mice) or MIC β1KO lesions (dormant, pooled lesions from n = 6 mice), specifically in the epithelial tumor cell cluster. **h** Fluorescent IHC for OPN, p-Stat3, PanCK, and DAPI on MIC WT (n = 8) and MIC Stat3KO (n = 6) mammary glands 2 weeks post-DOX induction. **i-j** Quantification of OPN+ cells and OPN+ PanCK+ cells in MIC WT (n = 8) and MIC Stat3KO (n = 6) mammary glands 2 weeks post-DOX induction. Scale bars are as indicated on each image. Mean ± SEM for data calculated using two-tailed Student's t test. Each data point is representative of one biological sample for (**b**), (**c**), (**e**), (**f**), (**i**), and (**j**). Source data are provided as a Source Data file.

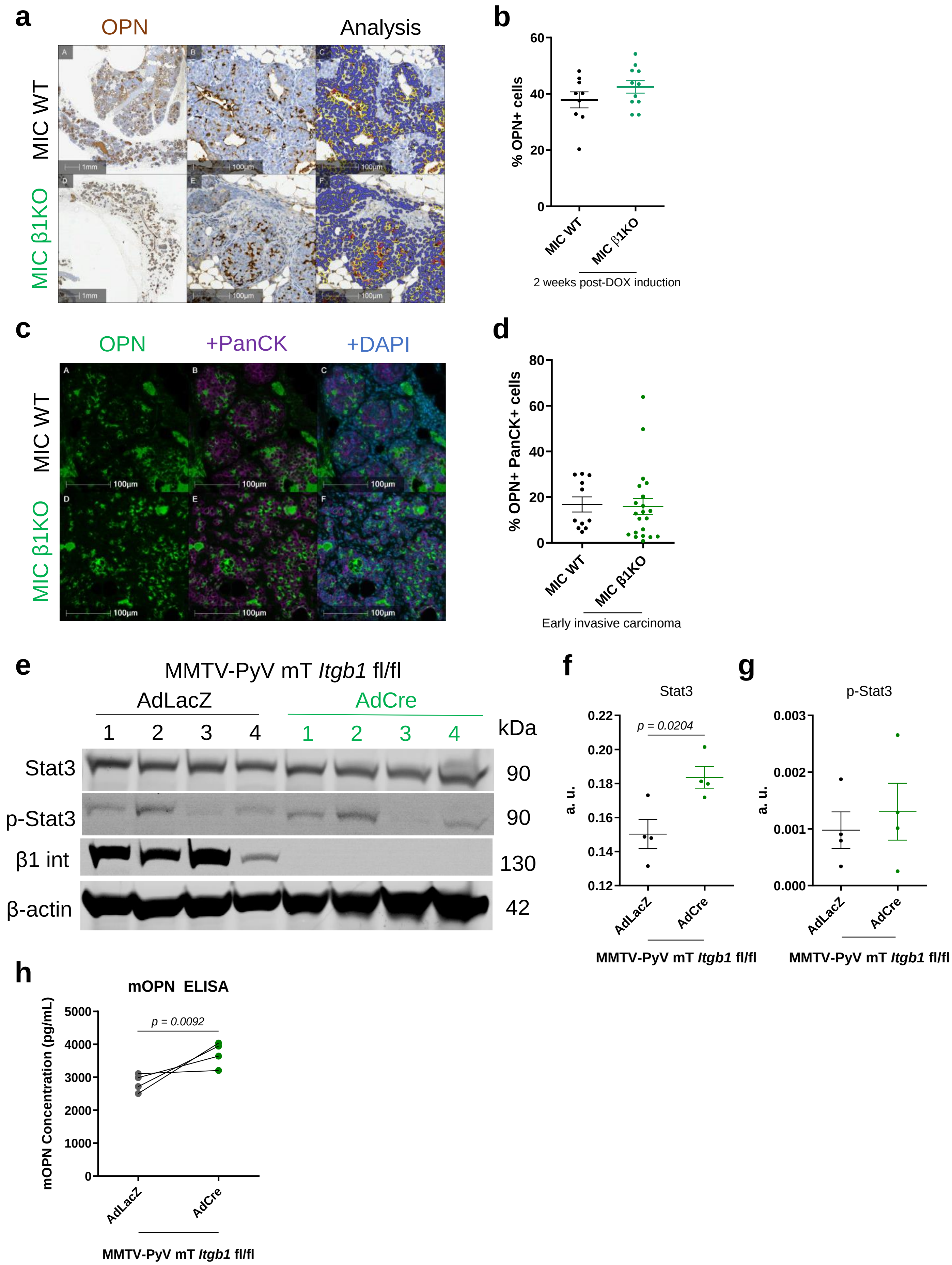
a
OPN
Analysis
MIC WT
MIC β1KO
1mm
100µm
b
% OPN+ cells
MIC WT
MIC β1KO
2 weeks post-DOX induction
c
OPN
+PanCK
+DAPI
MIC WT
MIC β1KO
100µm
d
% OPN+ PanCK+ cells
MIC WT
MIC β1KO
Early invasive carcinoma
e
MMTV-PyV mT Itgb1 fl/fl
AdLacZ
AdCre
1 2 3 4
1 2 3 4
kDa
Stat3 90
p-Stat3 90
β1 int 130
β-actin 42
f
Stat3
a. u.
p = 0.0204
AdLacZ
AdCre
MMTV-PyV mT Itgb1 fl/fl
g
p-Stat3
a. u.
AdLacZ
AdCre
MMTV-PyV mT Itgb1 fl/fl
h
mOPN ELISA
mOPN Concentration (pg/mL)
p = 0.0092
AdLacZ
AdCre
MMTV-PyV mT Itgb1 fl/fl

Supplementary Figure 2. OPN levels during different stages of MIC WT and MIC β1KO tumor progression and Stat3 and OPN levels after acute β1 integrin ablation.

a IHC staining for OPN on mammary glands of MIC WT (n = 9) and MIC β1KO (n = 11) mice 2 weeks post-DOX induction (early tumor initiation stage). **b** Quantification of OPN+ cells in mammary glands of MIC WT (n = 9) and MIC β1KO (n = 11) mice 2 weeks post-DOX induction. **c** Fluorescent IHC for OPN, PanCK, and DAPI on early invasive carcinoma from MIC WT lesions (fast-growing, n = 11) or MIC β1KO lesions (dormant, n = 21). **d** Quantification of OPN+ PanCK+ cells in early invasive carcinoma from MIC WT lesions (fast-growing, n = 11) or MIC β1KO lesions (dormant, n = 21). **e** Immunoblot for Stat3, p-Stat3, and β1 integrin (β1 int) with loading control (β-actin) on n = 4 MMTV-PyV mT cell lines carrying conditional floxed alleles (*Itgb1* fl/fl) at day 4 post-AdLacZ or AdCre viral infection. **f-g** Quantification of immunoblot for Stat3 and p-Stat3 normalized to β-actin (n = 4 cell lines). **h** Enzyme-linked immunosorbent assay (ELISA) for mouse OPN on cell culture media supernatant (diluted 1:20) of n = 4 MMTV-PyV mT cell lines carrying conditional floxed alleles (*Itgb1* fl/fl) at day 4 post-AdLacZ or AdCre viral infection. Scale bars are as indicated on each image. Mean ± SEM for data calculated using two-tailed Student's t test unless otherwise specified. Each data point is representative of one biological sample for (**b**) and (**d**) and one cell line for (**f-h**). Source data are provided as a Source Data file.

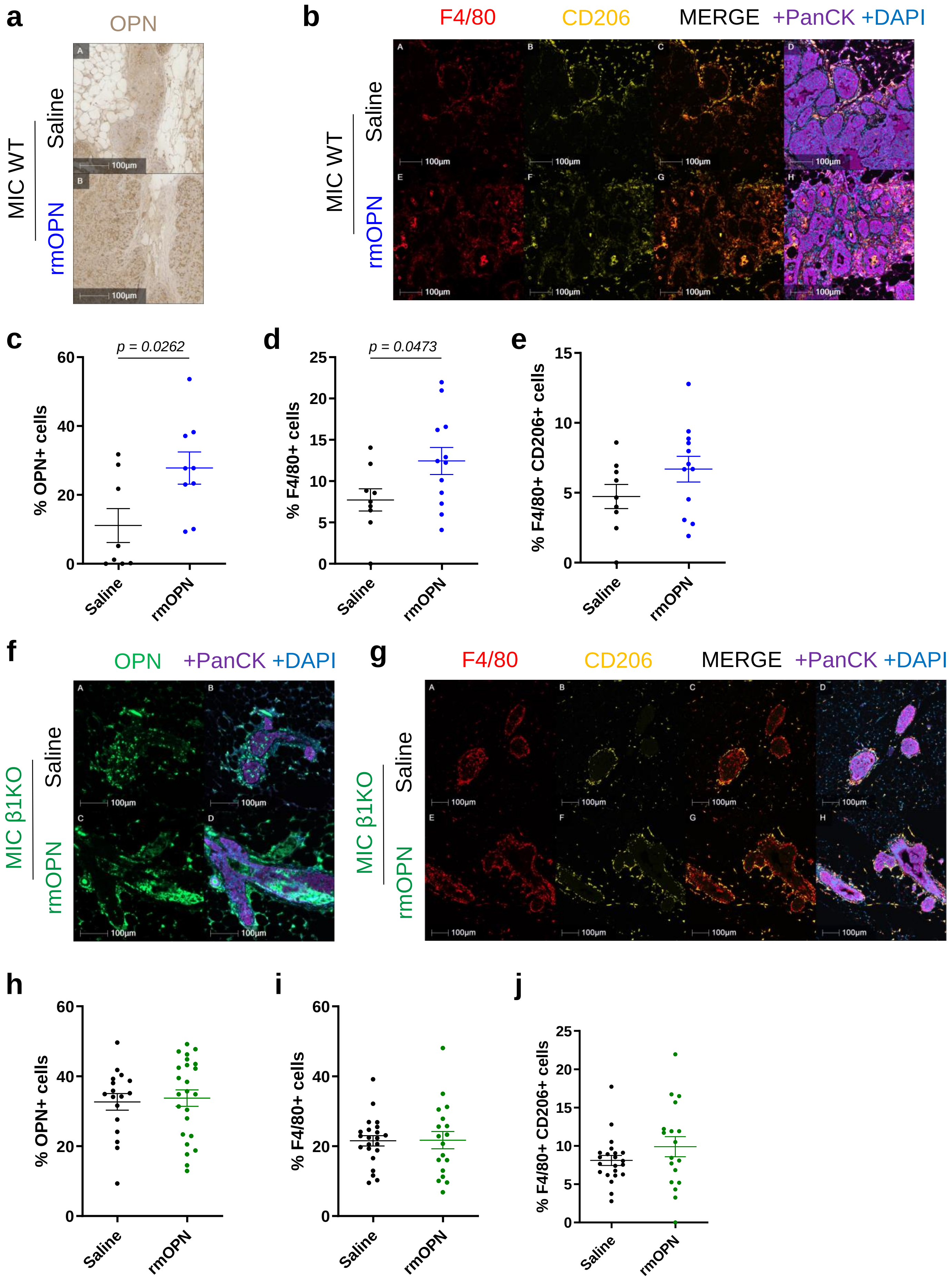
a
OPN
MIC WT
Saline
rmOPN
100µm
b
F4/80
CD206
MERGE
+PanCK +DAPI
MIC WT
Saline
rmOPN
100µm
c
p = 0.0262
% OPN+ cells
0
20
40
60
Saline
rmOPN
d
p = 0.0473
% F4/80+ cells
0
5
10
15
20
25
Saline
rmOPN
e
% F4/80+ CD206+ cells
0
5
10
15
Saline
rmOPN
f
OPN
+PanCK +DAPI
MIC β1KO
Saline
rmOPN
100µm
g
F4/80
CD206
MERGE
+PanCK +DAPI
MIC β1KO
Saline
rmOPN
100µm
h
% OPN+ cells
0
20
40
60
Saline
rmOPN
i
% F4/80+ cells
0
20
40
60
Saline
rmOPN
j
% F4/80+ CD206+ cells
0
5
10
15
20
25
Saline
rmOPN

Supplementary Figure 3. Characterization of the mammary tissue microenvironments of MIC WT and β1KO mice treated with recombinant mouse OPN.

a IHC for OPN on tumors and mammary glands of MIC WT mice treated with saline (n = 8) or recombinant mouse OPN (rmOPN) (n = 9). **b** Fluorescent IHC for F4/80, CD206, PanCK, and DAPI on tumors and mammary glands of MIC WT mice treated with saline (n = 9) or rmOPN (n = 12). **c-e** Quantification of OPN+ cells, F4/80+ cells, and F4/80+ CD206+ cells in tumors and mammary glands of MIC WT mice treated with saline or rmOPN. **f** Fluorescent IHC for OPN, PanCK, and DAPI on tumors and mammary glands of MIC β1KO mice treated with saline (n = 17) or rmOPN (n = 24). **g** Fluorescent IHC for F4/80, CD206, PanCK, and DAPI on tumors and mammary glands of MIC β1KO mice treated with saline (n = 22) or rmOPN (n = 18). **h-j** Quantification of OPN+ cells, F4/80+ cells, and F4/80+ CD206+ cells in tumors and mammary glands of MIC β1KO mice treated with saline or rmOPN. Scale bars are as indicated on each image. Mean ± SEM for data calculated using two-tailed Student's t test. Each data point is representative of one biological sample for (**c-e**) and (**h-j**). Source data are provided as a Source Data file.

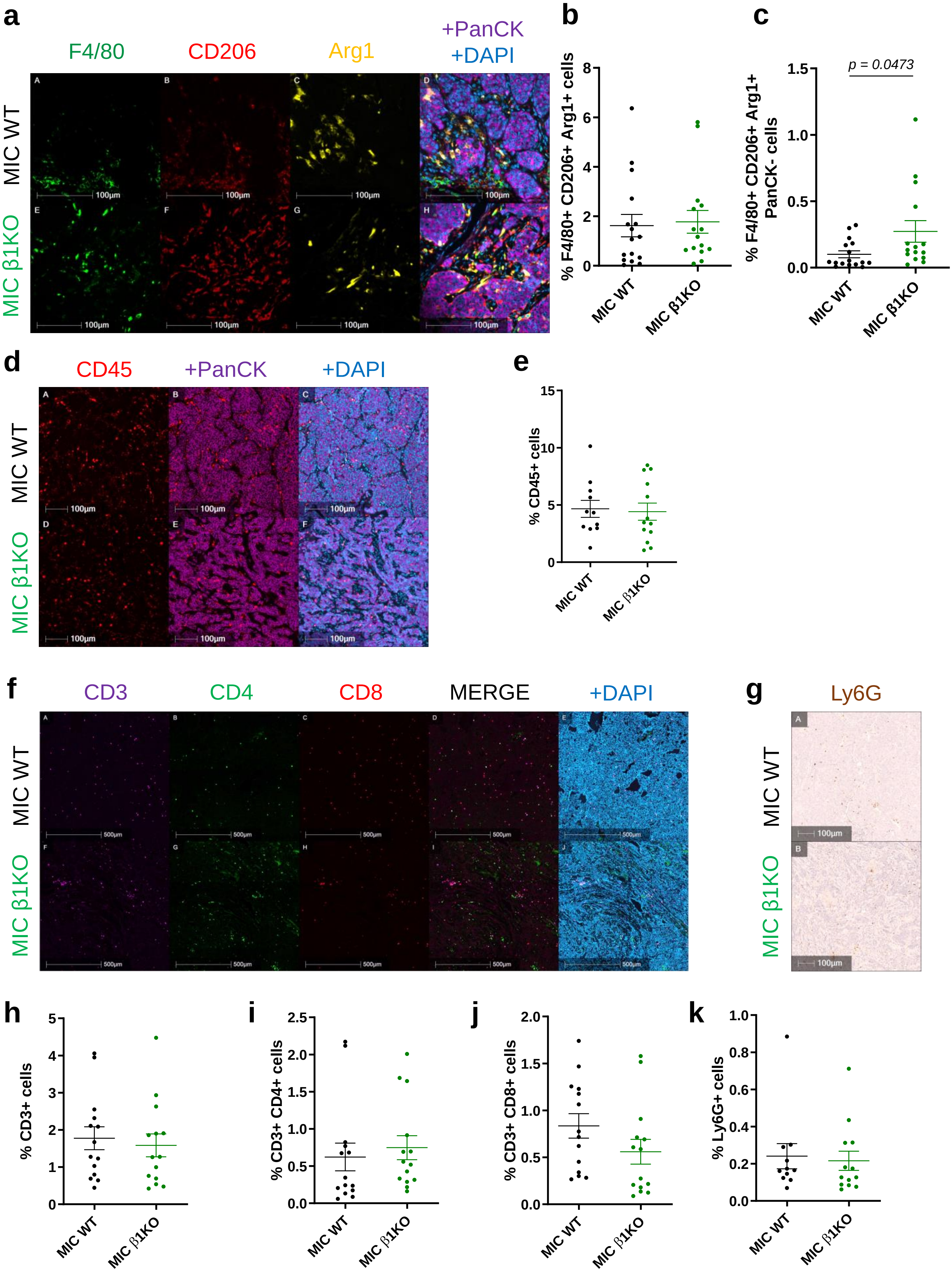

a
F4/80
CD206
Arg1
+PanCK
+DAPI
MIC WT
MIC β1KO
100µm
b
% F4/80+ CD206+ Arg1+ cells
MIC WT
MIC β1KO
c
% F4/80+ CD206+ Arg1+ PanCK- cells
p = 0.0473
MIC WT
MIC β1KO
d
CD45
+PanCK
+DAPI
MIC WT
MIC β1KO
e
% CD45+ cells
MIC WT
MIC β1KO
f
CD3
CD4
CD8
MERGE
+DAPI
MIC WT
MIC β1KO
500µm
g
Ly6G
MIC WT
MIC β1KO
h
% CD3+ cells
i
% CD3+ CD4+ cells
j
% CD3+ CD8+ cells
k
% Ly6G+ cells
MIC WT
MIC β1KO

Supplementary Figure 4. β1 integrin-deficient recurrent tumors have comparable levels of total immune cells, T cells, and neutrophils as wild-type tumors.

a Fluorescent IHC for F4/80, CD206, arginase 1 (Arg1), PanCK, and DAPI on MIC WT (n = 16) and MIC β1KO (n = 15) recurrent tumors. **b-c** Quantification of F4/80+ CD206+ Arg1+ cells and F4/80+ CD206+ Arg1+ PanCK- cells in MIC WT (n = 16) and MIC β1KO (n = 15) recurrent tumors. **d** Fluorescent IHC for CD45, PanCK, and DAPI on MIC WT (n = 11) and MIC β1KO (n = 13) recurrent tumors. **e** Quantification of CD45+ cells (total immune cells) in MIC WT (n = 11) and MIC β1KO (n = 13) recurrent tumors. **f** Fluorescent IHC for CD3, CD4, CD8, PanCK, and DAPI on MIC WT (n = 14) and MIC β1KO (n = 14) recurrent tumors. **g** IHC for Ly6G on MIC WT (n = 11) and MIC β1KO (n = 13) recurrent tumors. **h-k** Quantification of CD3+ cells (total T cells), CD3+ CD4+ cells (T helper cells), CD3+ CD8+ (cytotoxic T cells), and Ly6G+ (neutrophils) in the tumor in MIC WT and MIC β1KO recurrent tumors. Scale bars are as indicated on each image. Each data point is representative of one biological sample for (**b-c**), (**e**), and (**h-k**). Mean ± SEM for data calculated using two-tailed Student's t test. Source data are provided as a Source Data file.

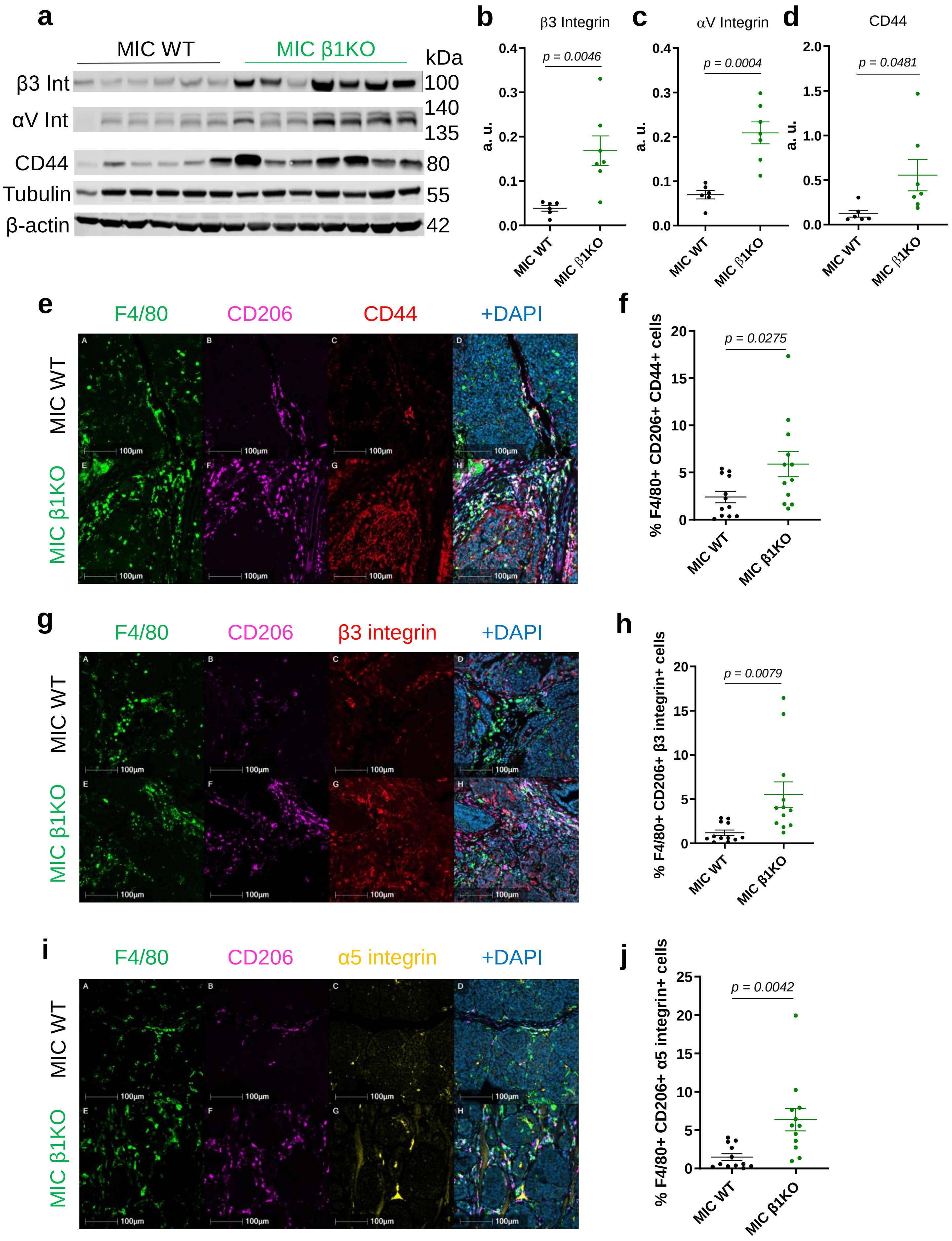

a
MIC WT
MIC β1KO
kDa
β3 Int
αV Int
CD44
Tubulin
β-actin
100
140
135
80
55
42
b
β3 Integrin
p = 0.0046
c
αV Integrin
p = 0.0004
d
CD44
p = 0.0481
a. u.
MIC WT
MIC β1KO
e
F4/80
CD206
CD44
+DAPI
f
p = 0.0275
% F4/80+ CD206+ CD44+ cells
g
F4/80
CD206
β3 integrin
+DAPI
h
p = 0.0079
% F4/80+ CD206+ β3 integrin+ cells
i
F4/80
CD206
α5 integrin
+DAPI
j
p = 0.0042
% F4/80+ CD206+ α5 integrin+ cells
100µm

Supplementary Figure 5. Pro-tumorigenic macrophages in β1 integrin-deficient recurrent tumors have higher levels of osteopontin receptors.

a Immunoblot for OPN receptors (β3 integrin, αV integrin, and CD44) with loading controls (β-actin and tubulin) on MIC WT (n = 6) and MIC β1KO (n = 7) recurrent tumor lysates. **b-d** Quantification of immunoblots for β3 integrin, αV integrin, and CD44 normalized to β-actin or to tubulin. **e** Fluorescent IHC for F4/80, CD206, CD44, and DAPI on MIC WT (n = 12) and MIC β1KO (n = 12) recurrent tumors. **f** Quantification of the percentage F4/80+ CD206+ CD44+ cells (OPN receptor-expressing pro-tumorigenic macrophages) in MIC WT (n = 12) and MIC β1KO (n = 12) recurrent tumors. **g** Fluorescent IHC for F4/80, CD206, β3 integrin, and DAPI on MIC WT (n = 12) and MIC β1KO (n = 12) recurrent tumors. **h** Quantification of the percentage of F4/80+ CD206+ β3 integrin+ cells (OPN receptor-expressing pro-tumorigenic macrophages) in MIC WT (n = 12) and MIC β1KO (n = 12) recurrent tumors. **i** Fluorescent IHC for F4/80, CD206, α5 integrin, and DAPI on MIC WT (n = 12) and MIC β1KO (n = 12) recurrent tumors. **j** Quantification of the percentage of F4/80+ CD206+ α5 integrin+ cells (OPN receptor-expressing pro-tumorigenic macrophages) in MIC WT (n = 12) and MIC β1KO (n = 12) recurrent tumors. Scale bars are as indicated on each image. Mean ± SEM for data calculated using two-tailed Student's t test. Each data point is representative of one biological sample for (**b-d**), (**f**), (**h**), and (**j**). Source data are provided as a Source Data file.

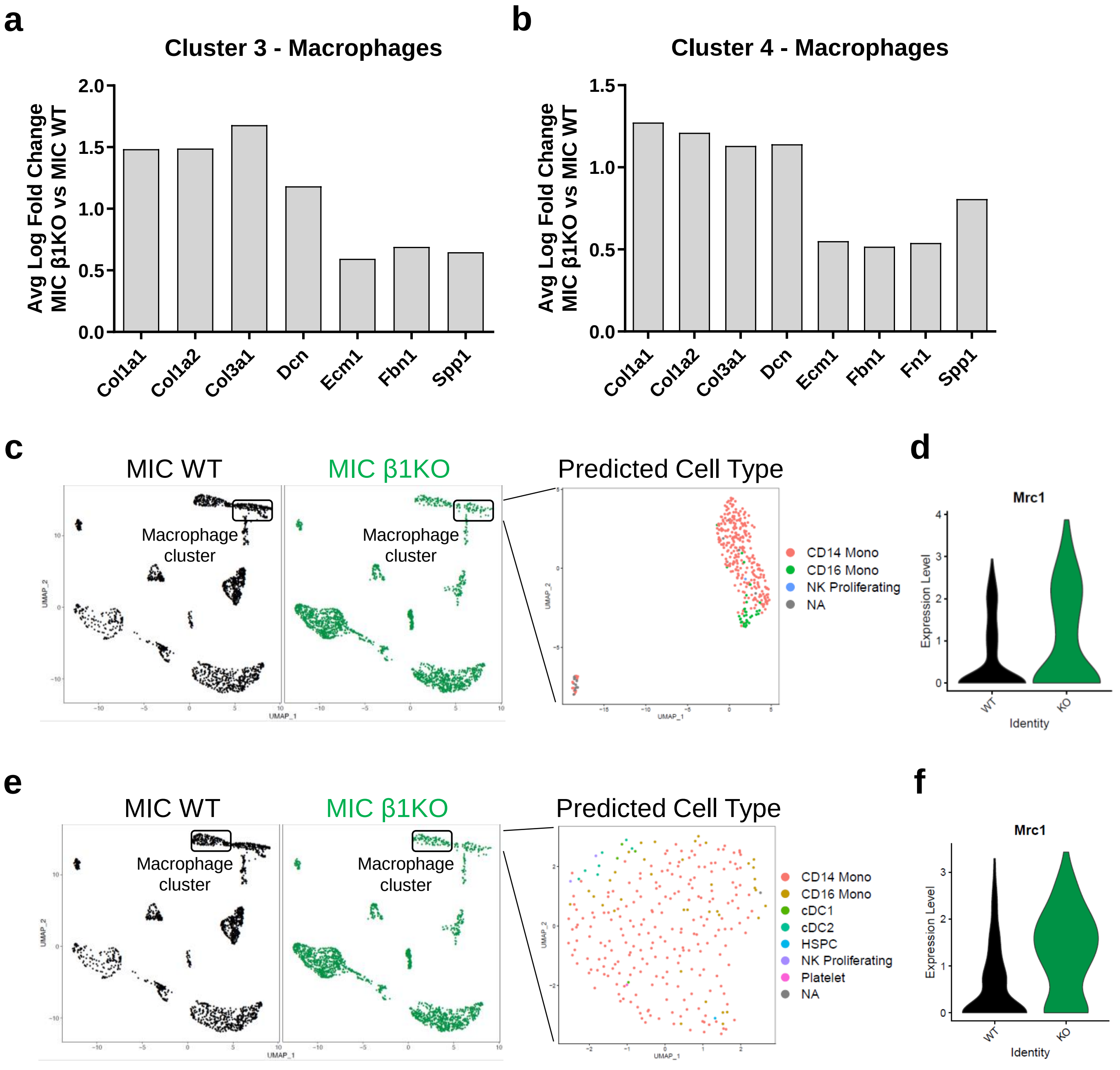

Supplementary Figure 6. Pro-tumorigenic macrophages upregulate extracellular matrix transcription contributing to tumor fibrosis during recurrence.
a-b Average log fold change of extracellular matrices in clusters 3 and 4 (macrophages) from single-cell RNA sequencing between MIC WT lesions (fast-growing) or MIC β1KO lesions (dormant), where the fold change above 0 reflects higher expression in MIC β1KO (dormant) lesions. **c** UMAP plots showing sub-clustering analysis of predicted cell types from the macrophage cluster 3 from single-cell RNA sequencing of early invasive tumors from MIC WT (fast-growing) or MIC β1KO (dormant) mice. **d** Violin plot showing *Mrc1* (CD206) expression, specifically in the macrophage cluster from **c**. **e** UMAP plots showing sub-clustering analysis of predicted cell types from the macrophage cluster 4 from single-cell RNA sequencing of early invasive tumors from MIC WT (fast-growing) or MIC β1KO (dormant) mice. **f** Violin plot showing *Mrc1* (CD206) expression, specifically in the macrophage cluster from **e**. Mean ± SEM for data calculated using two-tailed Student's t test. Source data are provided as a Source Data file.

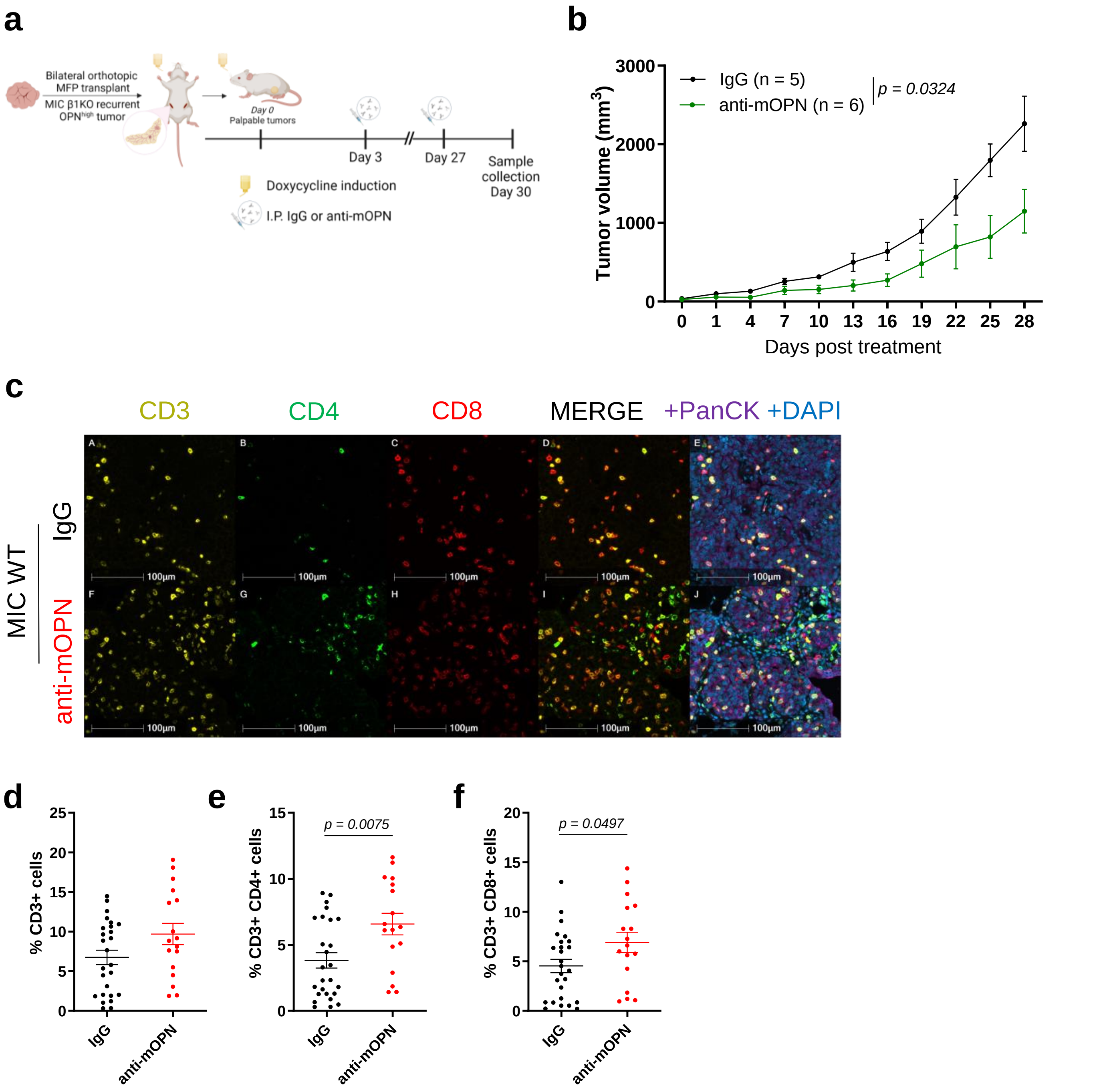

Supplementary Figure 7. Targeting osteopontin permits T cell-mediated tumor clearance. **a** Schematic representation of experimental design for FVB mice induced on doxycycline with mammary fat pad (MFP)-transplanted MIC β1KO recurrent tumors and treated for intraperitoneal injection of control antibody IgG or anti-mOPN. Created in BioRender. Muller, W. (2024) BioRender.com/x91n317. **b** Tumor volume measured from weekly palpations of FVB mice induced on doxycycline with MFP-transplanted MIC β1KO recurrent tumors treated with IgG or anti-mOPN. Two-tailed Students' t-test was performed at endpoint and n denotes number of tumors per treatment arm. **c** Fluorescent IHC for CD3, CD4, CD8, PanCK, and DAPI on tumors and mammary glands of MIC WT mice treated with IgG (n = 26) or anti-mOPN (n = 17). **d-f** Quantification of CD3+ cells, CD3+ CD4+ cells, and CD3+ CD8+ cells in tumors and mammary glands of MIC WT mice treated with IgG (n = 26) or anti-mOPN (n = 17). Scale bars are as indicated on each image. Mean ± SEM for data calculated using two-tailed Student's t test unless otherwise specified. Each data point is representative of one biological sample for (**d-f**). Source data are provided as a Source Data file.

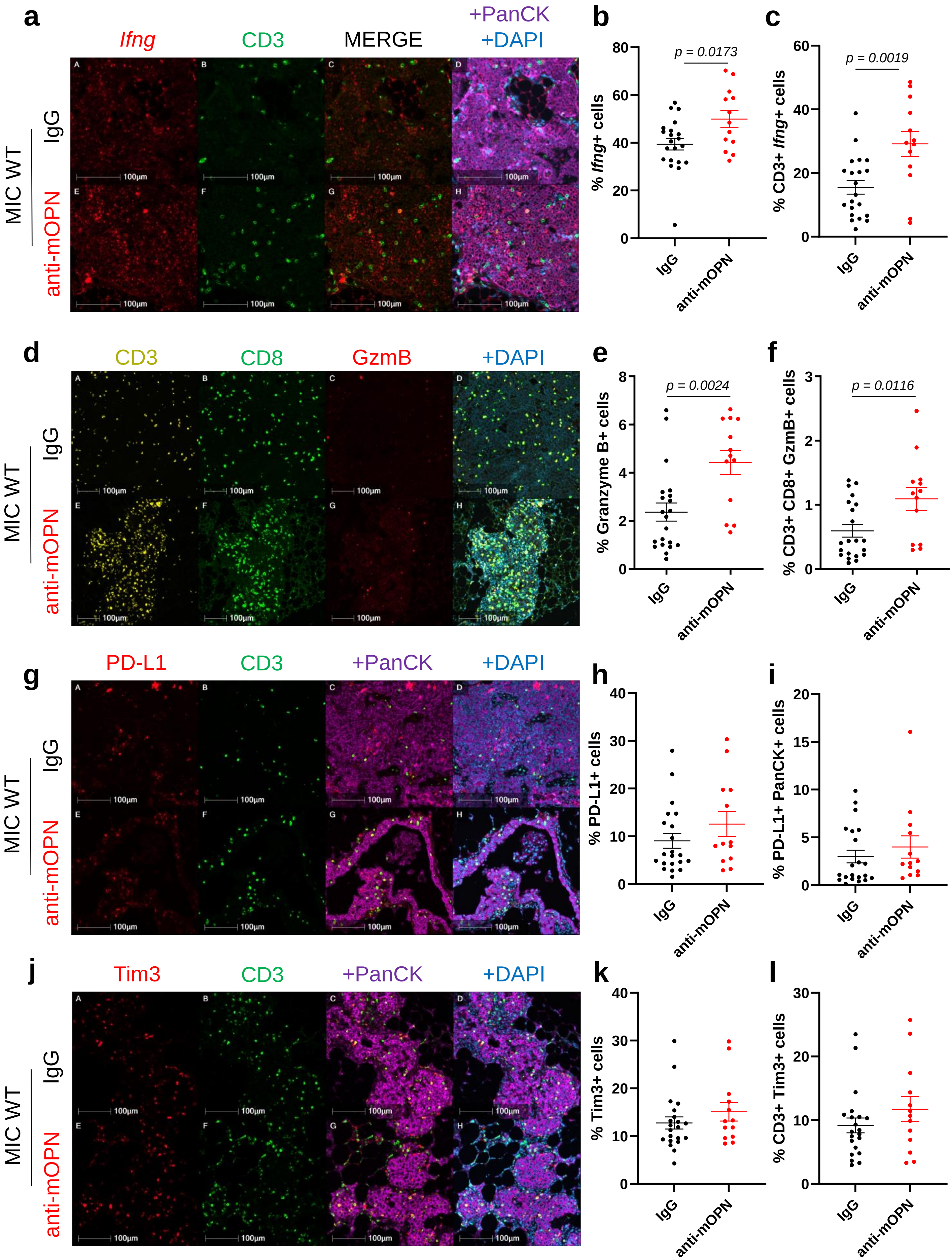

a
Ifng
CD3
MERGE
+PanCK
+DAPI
MIC WT
IgG
anti-mOPN
100µm
b
% Ifng+ cells
p = 0.0173
IgG
anti-mOPN
c
% CD3+ Ifng+ cells
p = 0.0019
d
CD3
CD8
GzmB
+DAPI
e
% Granzyme B+ cells
p = 0.0024
f
% CD3+ CD8+ GzmB+ cells
p = 0.0116
g
PD-L1
CD3
+PanCK
+DAPI
h
% PD-L1+ cells
i
% PD-L1+ PanCK+ cells
j
Tim3
CD3
+PanCK
+DAPI
k
% Tim3+ cells
l
% CD3+ Tim3+ cells

Supplementary Figure 8. T cell activation and exhaustion characterization anti-mOPN-treated mice.
a RNA Scope for mouse *Ifng* and fluorescent IHC for CD3 and DAPI on tumors and mammary glands of MIC WT mice treated with IgG or anti-mOPN. **b-c** Quantification of total *Ifng*+ cells and CD3+ *Ifng*+ cells in tumors and mammary glands of MIC WT mice treated with IgG or anti-mOPN. **d** Fluorescent IHC for CD3, CD8, Granzyme B (GzmB), and DAPI on tumors and mammary glands of MIC WT mice treated with IgG or anti-mOPN. **e-f** Quantification of GzmB+ cells and CD3+ GzmB+ cells in tumors and mammary glands of MIC WT mice treated with IgG or anti-mOPN. **g** Fluorescent IHC for PD-L1, CD3, PanCK, and DAPI on tumors and mammary glands of MIC WT mice treated with IgG or anti-mOPN. **h-i** Quantification of PD-L1+ cells and of PD-L1+ PanCK+ cells in tumors and mammary glands of MIC WT mice treated with IgG or anti-mOPN. **j** Fluorescent IHC for Tim3, CD3, PanCK, and DAPI on tumors and mammary glands of MIC WT mice treated with IgG or anti-mOPN. **k-l** Quantification of Tim3+ cells and CD3+ Tim3+ cells in tumors and mammary glands of MIC WT mice treated with IgG or anti-mOPN. Scale bars are as indicated on each image. Mean ± SEM for data calculated using two-tailed Student's t test. Each data point is representative of one biological sample for (**b-c**), (**e-f**), (**h-i**), and (**k-l**) with IgG (n = 21) and anti-mOPN (n = 13) for all. Source data are provided as a Source Data file.

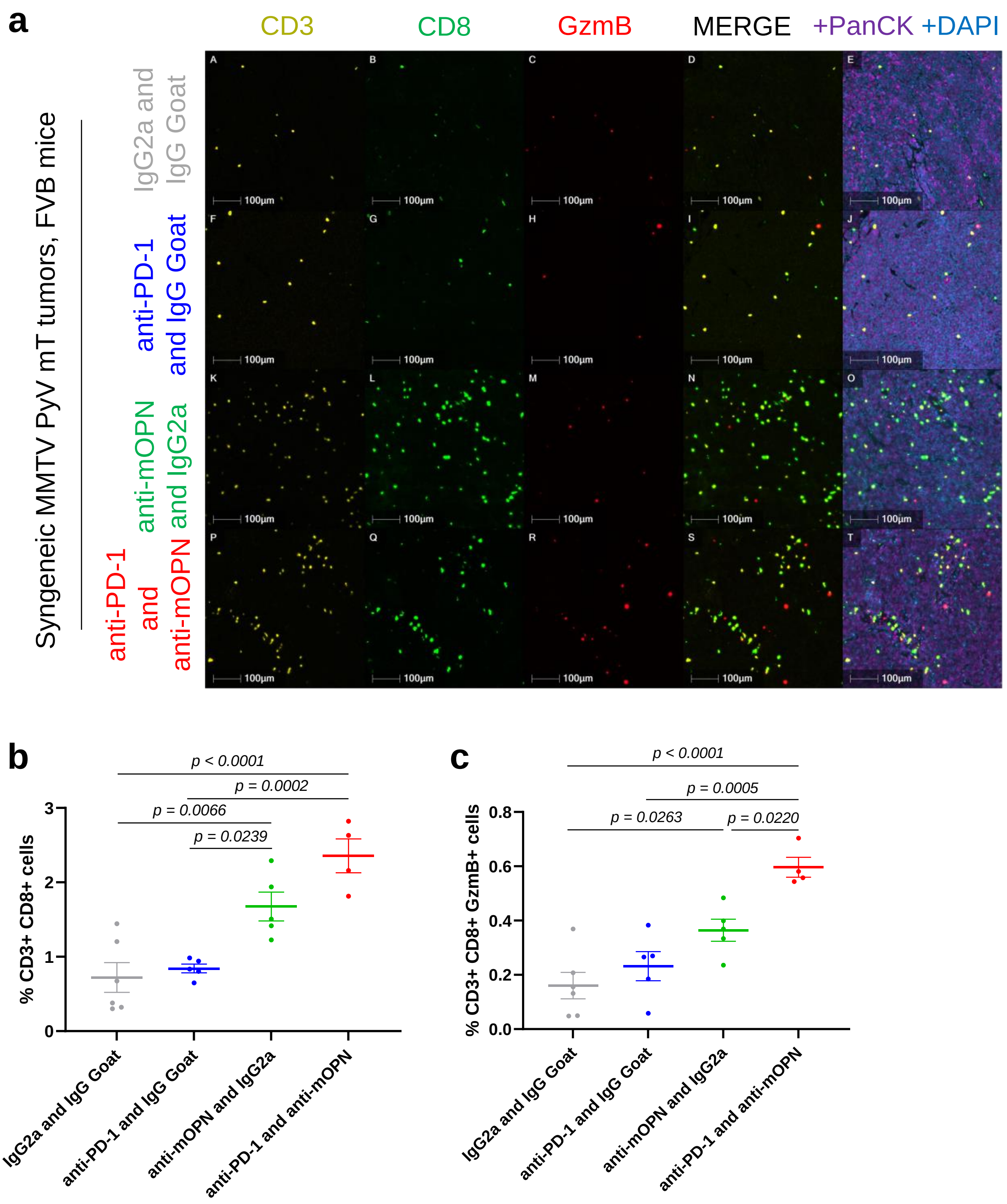

Supplementary Figure 9. Anti-mOPN and anti-PD-1 combinational treatment increases T cell infiltration and activation.
a Fluorescent IHC for CD3, CD8, granzyme B (GzmB) PanCK, and DAPI on syngeneic MMTV PyV mT tumors treated with IgG2a and IgG Goat (n = 6), anti-PD-1 and IgG Goat (n = 5), anti-mOPN and IgG2a (n = 5), or anti-PD-1 and anti-mOPN (n = 4). **b-c** Quantification of CD3+ CD8+ cells and CD3+ CD8+ GzmB+ cells in syngeneic MMTV PyV mT tumors treated with IgG2a and IgG Goat (n = 6), anti-PD-1 and IgG Goat (n = 5), anti-mOPN and IgG2a (n = 5), or anti-PD-1 and anti-mOPN (n = 4). Scale bars are as indicated on each image. Mean ± SEM for data calculated using Ordinary One Way ANOVA with Tukey's post hoc test. Each data point is representative of one biological sample for (**b-c**). Source data are provided as a Source Data file.

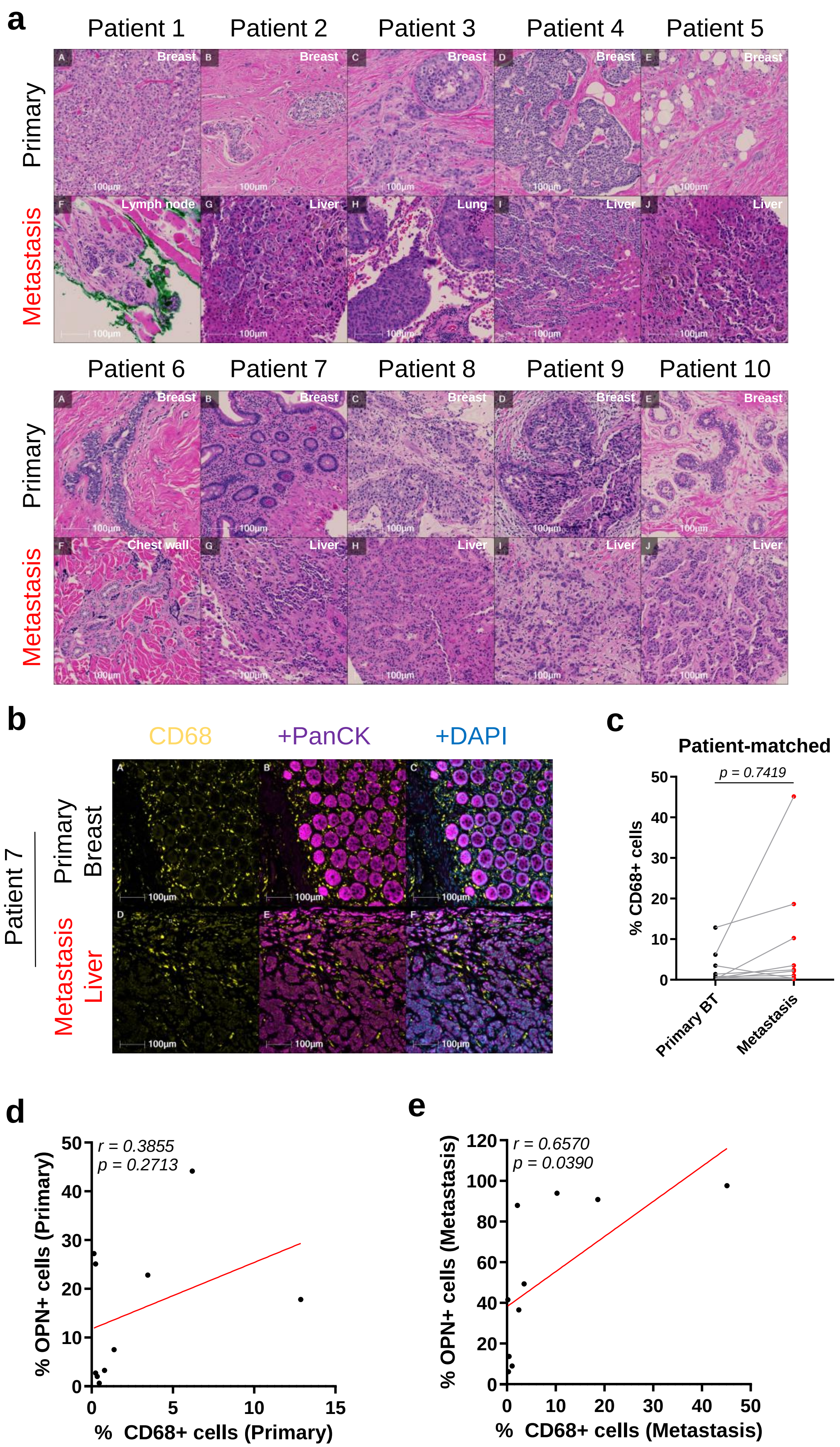
a
Patient 1
Patient 2
Patient 3
Patient 4
Patient 5
Primary
Metastasis
Breast
Lymph node
Liver
Lung
Liver
Liver
Patient 6
Patient 7
Patient 8
Patient 9
Patient 10
Primary
Metastasis
Breast
Chest wall
Liver
Liver
Liver
Liver
100μm
b
CD68
+PanCK
+DAPI
Patient 7
Primary Breast
Metastasis Liver
100μm
c
Patient-matched
p = 0.7419
% CD68+ cells
Primary BT
Metastasis
d
r = 0.3855
p = 0.2713
% OPN+ cells (Primary)
% CD68+ cells (Primary)
e
r = 0.6570
p = 0.0390
% OPN+ cells (Metastasis)
% CD68+ cells (Metastasis)

Supplementary Figure 10. Osteopontin positivity correlates with macrophage infiltration in patient-matched recurrent metastatic tumors.

a H&E image of patient-matched primary breast tumors and recurrent metastatic tumors. **b** Fluorescent IHC for CD68, PanCK, and DAPI on patient-matched primary breast tumors and recurrent metastatic samples. **c** Quantification of CD68+ cells in patient-matched primary breast tumors (n = 10) and recurrent metastatic tumors (n = 10). **d-e** Correlation between OPN+ cells and CD68+ cells in patient-matched primary breast tumors (n = 10) and recurrent metastatic tumors (n = 10). Statistical significance was calculated using the Spearman rank correlation test. Scale bars are as indicated on each image. Mean ± SEM for data calculated using two-tailed Student's t test unless otherwise specified. Each data point is representative of one biological sample for (**c-e**). Source data are provided as a Source Data file.

a

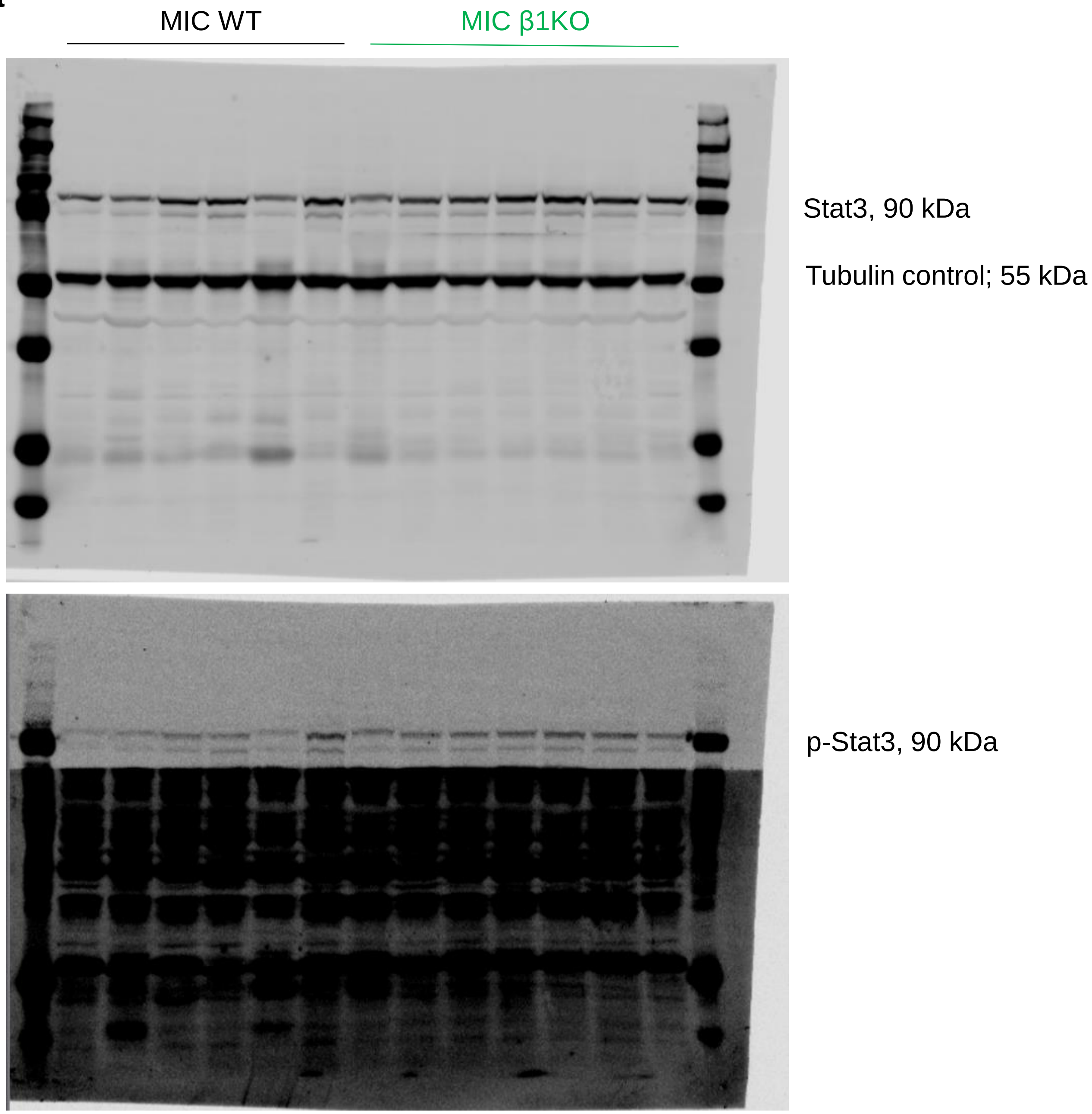

Supplementary Figure 11. Uncropped immunoblots for Supplementary Figure 1d.

a Immunoblot for Stat3, tubulin control, and p-Stat3 on MIC WT and MIC β1KO recurrent tumor lysates. The same immunoblot is presented twice for separate channels.

a

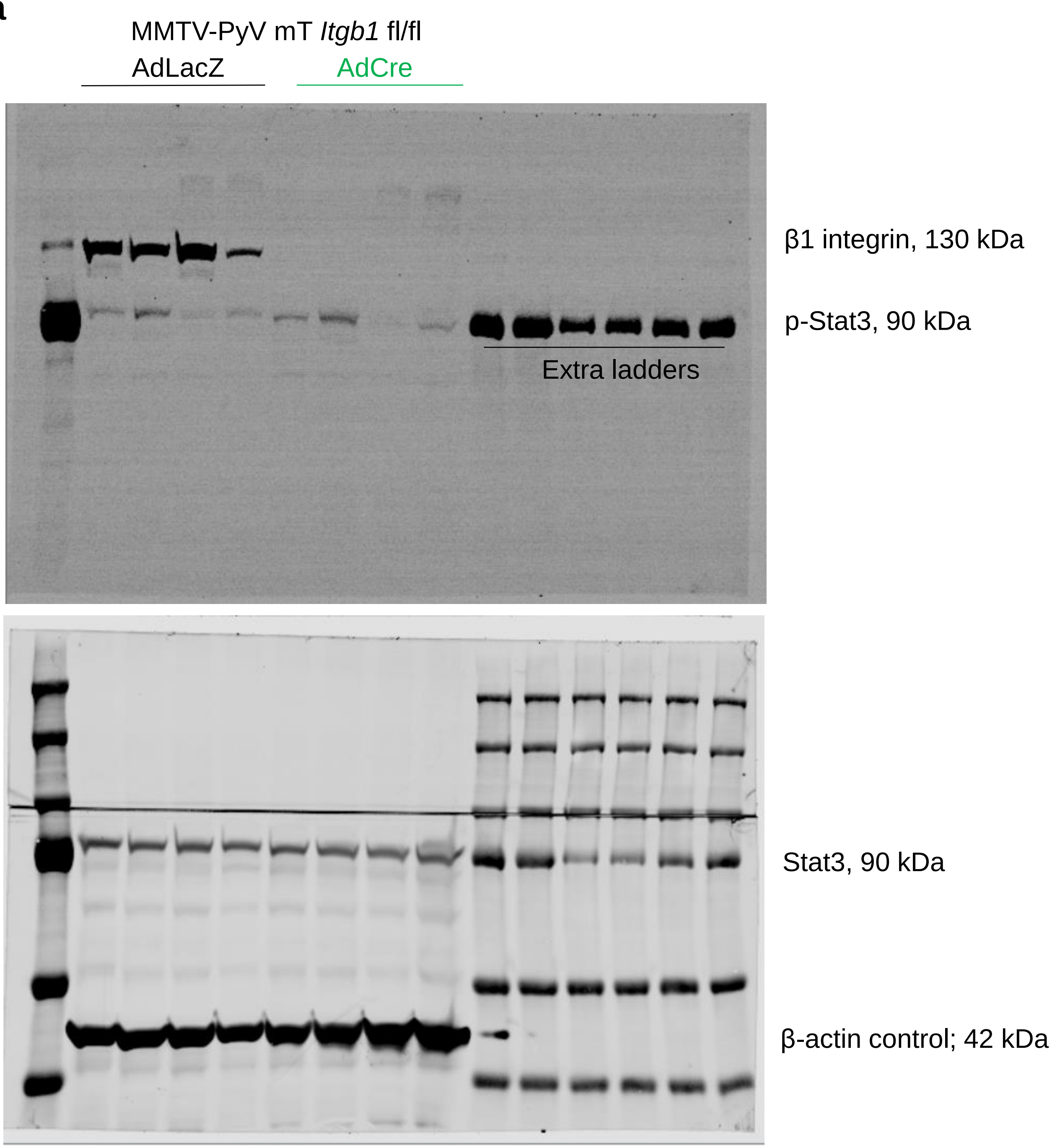

Supplementary Figure 12. Uncropped immunoblots for Supplementary Figure 2e.

a Immunoblot for β1 integrin and p-Stat3 and immunoblot for Stat3 and β-actin control on four MMTV-PyV mT cell lines carrying conditional floxed alleles (*Itgb1* fl/fl) at day 4 post-AdLacZ or AdCre viral infection. The same immunoblot is presented twice for separate channels.

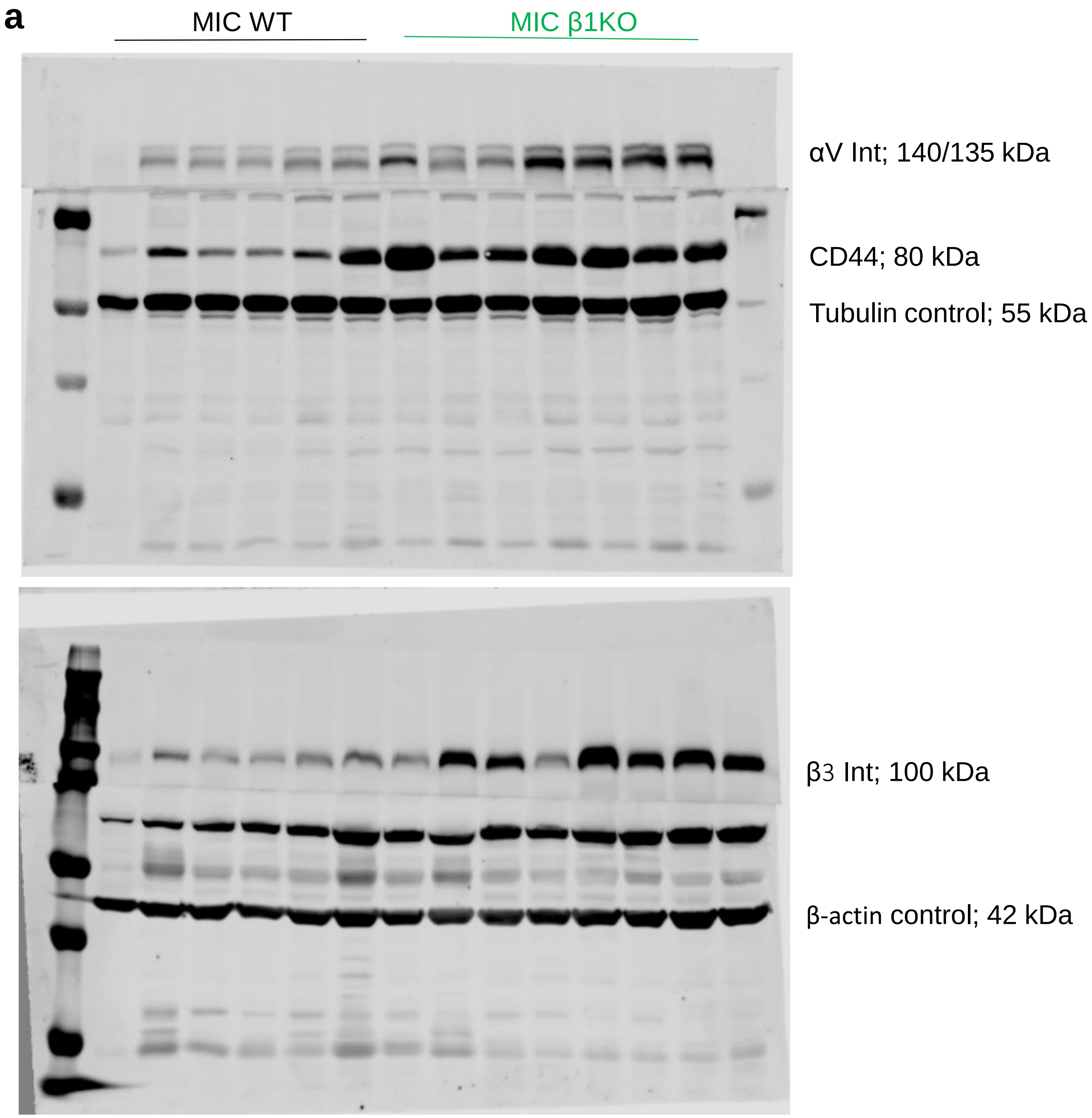

Supplementary Figure 13. Uncropped immunoblots for Supplementary Figure 5a.

a Immunoblot for OPN receptors αV integrin, CD44 and tubulin control and immunoblot for OPN receptors β3 integrin and β-actin control on MIC WT and MIC β1KO recurrent tumor lysates.
