# Supplementary material for: Osteopontin is a therapeutic target that drives breast cancer recurrence
Source: Nat Commun. 2024 Oct 24;15:9174. doi: 10.1038/s41467-024-53023-9 (PMC11502809; doi:10.1038/s41467-024-53023-9)
Supplement: Supplementary file 3 — Reporting Summary [file 41467_2024_53023_MOESM3_ESM.pdf]

nature portfolio

Corresponding author(s): William J. Muller

Last updated by author(s): Sep 5, 2024

Reporting Summary

Nature Portfolio wishes to improve the reproducibility of the work that we publish. This form provides structure for consistency and transparency in reporting. For further information on Nature Portfolio policies, see our Editorial Policies and the Editorial Policy Checklist.

Statistics

For all statistical analyses, confirm that the following items are present in the figure legend, table legend, main text, or Methods section.

n/a	Confirmed	
☐	☒	The exact sample size (n) for each experimental group/condition, given as a discrete number and unit of measurement
☐	☒	A statement on whether measurements were taken from distinct samples or whether the same sample was measured repeatedly
☐	☒	The statistical test(s) used AND whether they are one- or two-sided<br>*Only common tests should be described solely by name; describe more complex techniques in the Methods section.*
☒	☐	A description of all covariates tested
☐	☒	A description of any assumptions or corrections, such as tests of normality and adjustment for multiple comparisons
☐	☒	A full description of the statistical parameters including central tendency (e.g. means) or other basic estimates (e.g. regression coefficient) AND variation (e.g. standard deviation) or associated estimates of uncertainty (e.g. confidence intervals)
☐	☒	For null hypothesis testing, the test statistic (e.g. F, t, r) with confidence intervals, effect sizes, degrees of freedom and P value noted<br>*Give P values as exact values whenever suitable.*
☒	☐	For Bayesian analysis, information on the choice of priors and Markov chain Monte Carlo settings
☒	☐	For hierarchical and complex designs, identification of the appropriate level for tests and full reporting of outcomes
☐	☒	Estimates of effect sizes (e.g. Cohen's d, Pearson's r), indicating how they were calculated

Our web collection on statistics for biologists contains articles on many of the points above.

Software and code

Policy information about availability of computer code

Data collection

Tumor and lung metastasis: All solid organ tissues stined with H&E were scanned using Scanscope XT Digital Slide Scanner (Aperio Technologies) and analyzed using HALO 2.0 software (Indica Lab).

Fluorescent IHC and, RNA Scope: Stained slides were scanned using the Axio Scan Z1 digital slide scanner (Carl Zeiss) and analyzed using HALO 2.0 software (Indica Lab). RNAscope In-situ hybridization was performed using RNAscope® 2.5 HD Assay-RED kit (ACD, #322360) according to the manufacturer's protocol. The following probes were used: Mouse-Mm-Spp1 (Cat Number 435191), Mouse-IL-4 (Cat Number 312741), and Mouse-Mm-Ifng (Cat Number 311391). For Fig. 1D and 2D, this protocol was followed with fluorescent IHC for PanCK and DAPI.

RNA extraction and RT-qPCR: Flash-frozen pieces of tumors were crushed in liquid nitrogen. Total RNA was isolated using FavorPrep™ Tissue Total RNA Mini Kit (Cat Number FATRK 001) according to manufacturer's protocol. RNA quantity was determined using NanoDrop Spectrophotometer ND-1000 (NanoDrop Technologies, Inc.). cDNA was synthesized by reverse transcription using the TranScript all-in-one first strand cDNA synthesis kit (Transgen Biotech). Real-time qPCR was performed using LightCycler 480 SYBR Green I Master Reagents (Roche). Data were normalized to Gapdh to generate the relative transcript levels. Primer sequences are provided in the "Materials and Methods" section of the manuscript.

Immunoblot: Images were acquired using Li-Cor Odyssey Scanner. Band intensity quantification was done using Image Studio Lite software (Li-Cor).

Enzyme-linked immunosorbent assay (ELISA): The supernatant was collected 7 days post-infection and diluted 1:20. Osteopontin (OPN/SPP1) Mouse ELISA Kit (Invitrogen EMSPP1) was used according to manufacturer's protocol.

	Flow cytometry: BD LSR Fortessa flow cytometer and Flowjo 10.6.2 were used for data collection and analysis, respectively.<br><br>Transwell Migration Assay: Image acquisition was done on ZEISS Axio Zoom.V16 microscope (objective 30X). Cells in one representative field per image (not shown) was counted manually in Fig. 3C.<br><br>In vitro proliferation assay: MMTV-PyV mT cells were seeded in 96-well optical-bottomed plates (Nunc, 167008) at 8000 cells/well supplemented with recombinant mouse osteopontin at 1.0, 5.0 or 25.0 µg/mL or bovine serum albumin (BSA) in triplicates. IncuCyte S3 system (ESSEN BioSciences) was used for live cell imaging at 10X for 2 images/well, every 4 hours for 60 hours. The confluence percentage was calculated using the IncuCyte S3 analysis software.
Data analysis	Statistical analyses were performed on Prism 9.0 (Broad Institute, v4.i0), Kaplan-Meier survival curves (https://kmplot.com/analysis/), HALO Software Image Analysis (Indica Labs), Image Studio Lite (v5.2.1, Li-COR Biosciences), Microsoft Excel (v16.66.1, Microsoft), and IncuCyte S3 system (ESSEN BioSciences), TIMER2.0 (http://timer.comp-genomics.org/timer/), and BioRender was used to created schematic diagrams and figures (https://www.biorender.com/). More details are provided in the manuscript and figures.

For manuscripts utilizing custom algorithms or software that are central to the research but not yet described in published literature, software must be made available to editors and reviewers. We strongly encourage code deposition in a community repository (e.g. GitHub). See the Nature Portfolio guidelines for submitting code & software for further information.

Data

Policy information about availability of data

All manuscripts must include a data availability statement. This statement should provide the following information, where applicable:

- Accession codes, unique identifiers, or web links for publicly available datasets
- A description of any restrictions on data availability
- For clinical datasets or third party data, please ensure that the statement adheres to our policy

Single-cell RNA sequencing data and bulk RNA sequencing data that support the findings of this study have been deposited in NCBI GEO with the accession codes GSE186118 [https://www.ncbi.nlm.nih.gov/geo/query/acc.cgi?acc=GSE186118] and GSE186491 [https://www.ncbi.nlm.nih.gov/geo/query/acc.cgi?acc=GSE186491], respectively. All human primary breast cancer datasets are publicly available and obtained from NCBI GEO or the authors' websites. Respective NCBI GEO accession codes are indicated in their respective figure legends. No codes were generated for this study. Source Data provided with this paper. Analysis for Fig. 9a is based on the publicly available GEO database GSE58644 [https://www.ncbi.nlm.nih.gov/geo/query/acc.cgi?acc=GSE58644]. Forest plot analysis for Fig. 9B is based on publicly available datasets as indicated in each study on the figure and performed as per study by Abravanel et al [https://www.ncbi.nlm.nih.gov/pmc/articles/PMC4497740/]. Analysis for Fig. 9C is based on the publicly available GEO database GSE9014 [https://www.ncbi.nlm.nih.gov/geo/query/acc.cgi?acc=GSE9014].

Research involving human participants, their data, or biological material

Policy information about studies with human participants or human data. See also policy information about sex, gender (identity/presentation), and sexual orientation and race, ethnicity and racism.

Reporting on sex and gender	This study takes into consideration that breast cancer predominantly affects women although we acknowledge that 1% of cases occur in men. Therefore, our study solely uses female experimental mice to recapitulate breast cancer in women (biological attribute). All patient samples in this study are from women.
Reporting on race, ethnicity, or other socially relevant groupings	This study does not take into account race, ethnicity, or other socially relevant groupings.
Population characteristics	Tissue Micro arrays (TMA) of human breast cancer: TMAs were obtained from US Biomax Inc (BR1504b trial and BC081120f trial).<br><br>Patient-matched primary breast tumor samples and recurrent metastatic tumor samples included FFPE tissues of ER positive/HER2 negative primary treatment-naïve breast cancers and matched metastatic tissue samples.
Recruitment	This study does not involve recruitment.
Ethics oversight	Patient-matched primary breast tumor samples and recurrent metastatic tumor samples were obtained with IRB approval (Dana Farber/Harvard Cancer Center protocols 09-204 and 05-246) and with patient consent.

Note that full information on the approval of the study protocol must also be provided in the manuscript.

Field-specific reporting

Please select the one below that is the best fit for your research. If you are not sure, read the appropriate sections before making your selection.

☒ Life sciences ☐ Behavioural & social sciences ☐ Ecological, evolutionary & environmental sciences

For a reference copy of the document with all sections, see nature.com/documents/nr-reporting-summary-flat.pdf

Life sciences study design

All studies must disclose on these points even when the disclosure is negative.

Sample size	Sample sizes are indicated in each figure and figure legend. No power analysis or statistical method were used to calculate the sample size, they were determined based on our previous experimental designs and results. Data from all experiments were analyzed and p-values from statistical tests used to assess statistical significant and appropriateness of sample sizes.
Data exclusions	This study did not exclude any data from the analysis.
Replication	For all in vivo experiments, n is indicated for each in the manuscript for independent biological replicates.Multiple samples were obtained per experimental mouse at their respective experimental endpoints experiments and each data point indicates one replicate on graphs. All attempts are replication were successful when applicable. In vivo experiments were not replicated for logistic and ethical reasons. Replicates are shown in the figure and outlined in the figure legends.
Randomization	For all in vivo treatment experiments, experimental female mice were randomly assigned to a treatment arm.
Blinding	For all in vivo therapeutic treatment experiments, drug administration and tumor measurements were performed by different individuals, the latter blinded with respect to the treatment arms.

Reporting for specific materials, systems and methods

We require information from authors about some types of materials, experimental systems and methods used in many studies. Here, indicate whether each material, system or method listed is relevant to your study. If you are not sure if a list item applies to your research, read the appropriate section before selecting a response.

Materials & experimental systems

n/a	Involved in the study	
☐	☒	Antibodies
☐	☒	Eukaryotic cell lines
☒	☐	Palaeontology and archaeology
☐	☒	Animals and other organisms
☒	☐	Clinical data
☒	☐	Dual use research of concern
☒	☐	Plants

Methods

n/a	Involved in the study	
☒	☐	ChIP-seq
☐	☒	Flow cytometry
☒	☐	MRI-based neuroimaging

Antibodies

Antibodies used

Fluorescent IHC: The following antibodies were used for fluorescent IHC on mouse tissue: PanCK (Ventana, 760-2595, 1:10), OPN (Santa Cruz, sc-21472, 1:1200), p-Stat3 (Cell Signaling Technology (CST), 9145, 1:100), F4/80 (CST, 70076, 1:200), CD206 (CST, 24595, 1:400), CD45 (CST, 70257, 1:200), CD3 (Abcam, Ab16669, 1:200), CD4 (CST, 25229, 1:50), CD8 (CST, 98941, 1:200), CD44 (CST, 37259, 1:200), β3 integrin (CST, 13166, 1:200), Ki67 (Cell Signaling Technology CST, 12202, 1:200), PD-1 (CST, 84651, 1:200), arginase 1 (CST, 93668, 1:100), α5 integrin (Santa Cruz, sc-376199, 1:100), granzyme B (CST, 44153, 1:200), PD-L1 (CST, 64988, 1:100), and Tim3 (CST, 83882, 1:200). The following antibodies were used for fluorescent IHC on human tissue: PanCK (Ventana, 760-2595, 1:10), OPN (Abcam, Ab63856, 1:100), and CD68 (Ventana, 790-2931 1:5).

Immunoblot: The following antibodies were used for immunoblots: Stat3 (CST, 9139, 1:1000), p-Stat3 (CST, 9145, 1:1000), β-actin (Sigma, A5441, 1:2000), tubulin (CST, 2148, 1:1000), β3 integrin (Abcam, ab119992, 1:1000), αV integrin (CST, 60896, 1:1000) and CD44 (CST, 37259, 1:1000).

Flow cytometry: Fixed cells were stained for viability using the fixable viability dye eFluorTM 506 (ThermoFisher/eBioscience 65-0866-14), F4/80 (BD bioscience 123114), CD11b (Biolegend 563168), CD206 (Biolegend 141706), IL-4R (Biolegend 504117) and Arginase 1 (Invitrogen 46-3697-82).

In vivo experiments: The following antibodies were used for in vivo experiments: recombinant mouse osteopontin (R&D Systems 441-OP), osteopontin neutralizing antibody (R&D Systems AF808), anti-PD-1 (RMP1-14, Bio X cell #BE0146), Normal Goat IgG Control (R&D Systems AB-108-C), and Rat IgG2a (Bio X cell #BE0089).

Validation

The following antibodies were used for fluorescent IHC on mouse tissue:
• PanCK (Ventana, 760-2595, 1:10): Anti-Pan Keratin (AE1/AE3/PCK26) Primary Antibody is an antibody cocktail intended for laboratory use in the qualitative immunohistochemical detection of most acidic cytokeratins and all basic cytokeratins. Species Reactivity: Mouse.
• OPN (Santa Cruz, sc-21472, 1:1200): Mouse monoclonal IgG1K osteopontin/OPN/SPP1 antibody raised against mouse recombinant osteopontin for detection of OPN by WB, IP, IF and IHC(P). Product Clone Name: AKm2A1. Species Reactivity: Human, Mouse, Rat.
• p-Stat3 (Cell Signaling Technology (CST), 9145, 1:100): Phospho-Stat3 (Tyr705) (D3A7) XP(R) Rabbit monoclonal antibody detects endogenous levels of Stat3 only when phosphorylated at tyrosine 705. This antibody does not cross-react with phospho-EGFR or the

corresponding phospho-tyrosines of other Stat proteins. Species Reactivity: Human, Mouse, Rat, Monkey.
• F4/80 (CST, 70076, 1:200): Monoclonal antibody is produced by immunizing animals with recombinant mouse F4/80 protein.F4/80 (D2S9R) XP® Rabbit mAb recognizes endogenous levels of total F4/80 protein. Species Reactivity: Mouse.
• CD206 (CST, 24595, 1:400): Monoclonal antibody is produced by immunizing animals with a synthetic peptide corresponding to residues near the carboxy terminus of mouse CD206/MRC1 protein.CD206/MRC1 (E6T5J) XP® Rabbit mAb recognizes endogenous levels of total CD206/MRC1 protein. This antibody recognizes mouse CD206/MRC1 protein and is also reactive with human CD206/MRC1; however, this antibody is not suggested for immunohistochemical analysis of human tissues. Instead, CD206/MRC1 (E2L9N) Rabbit mAb #91992 is recommended for IHC analysis of human tissue samples. Species Reactivity: Human, Mouse, Rat, Monkey.
• CD45 (CST, 70257, 1:200): CD45 (D3F8Q) Rabbit mAb recognizes endogenous levels of total CD45 protein. Non-specific staining was observed in mouse kidney by immunohistochemistry. This antibody is predicted to react with both the CD45.1 and CD45.2 alleles. Species Reactivity: Mouse.
• CD3 (Abcam, Ab16669, 1:200): Anti-CD3 epsilon rabbit monoclonal antibody [SP7] targets the CD3 complex mediates signal transduction. This antibody is suitable for staining normal and neoplastic T cells in formalin-fixed, paraffinembedded tissues. Ab135372 is an alternative. Species Reactivity: Human, Mouse, Rat,
• CD4 (CST, 25229, 1:50): Monoclonal antibody is produced by immunizing animals with a synthetic peptide corresponding to residues surrounding Ala232 of mouse CD4 protein.CD4 (D7D2Z) Rabbit mAb recognizes endogenous levels of total mouse and rat CD4 protein. Non-specific staining in mouse kidney and liver has been observed. Species Reactivity: Mouse, Rat, Hamster.
• CD8 (CST, 98941, 1:200): Monoclonal antibody is produced by immunizing animals with a synthetic peptide corresponding to residues surrounding Asp42 of mouse CD8α protein.CD8α (D4W2Z) XP® Rabbit mAb recognizes endogenous levels of total CD8α protein. Species Reactivity: Mouse.
• CD44 (CST, 37259, 1:200): CD44 (E7K2Y) XP(R) Rabbit mAb recognizes endogenous levels of total CD44 protein. Monoclonal antibody is produced by immunizing animals with a synthetic peptide corresponding to residues surrounding Pro136 of human CD44 protein. This sequence region is conserved in all isoforms of CD44 reported in Uniprot, with the exception of isoform 2 and isoform 19. Species Reactivity: Human, Mouse, Rat.
• β3 integrin (CST, 13166, 1:200): Integrin β3 (D7X3P) XP(R) Rabbit mAb recognizes endogenous levels of total integrin β3 protein. Monoclonal antibody is produced by immunizing animals with a synthetic peptide corresponding to residues surrounding Ile114 of human integrin β3 protein. Species Reactivity: Human, Mouse.
• Ki67 (CST, 12202, 1:200): Ki-67 (D3B5) Rabbit mAb (IHC Formulated) recognizes endogenous levels of murine Ki-67 protein. It will also detect endogenous levels of human Ki-67 protein; however, Ki-67 (D2H10) Rabbit mAb #9027 is recommended for the detection of human Ki-67 protein in paraffin-embedded tissues. Species Reactivity: Mouse.
• PD-1 (CST, 84651, 1:200): PD-1 (Intracellular Domain) (D7D5W) XP® Rabbit mAb recognizes endogenous levels of total PD-1 protein. Monoclonal antibody is produced by immunizing animals with a synthetic peptide corresponding to residues surrounding Ala242 of mouse PD-1 protein. Species Reactivity: Mouse.
• Arginase 1 (CST, 93668, 1:100): Arginase-1 (D4E3M™) XP® Rabbit mAb recognizes endogenous levels of total arginase-1 protein. This antibody does not cross-react with arginase-2. Monoclonal antibody is produced by immunizing animals with a synthetic peptide corresponding to residues surrounding Val47 of human arginase-1 protein. Species Reactivity: Human, Mouse, Rat.
• α5 integrin (Santa Cruz, sc-376199, 1:100): Integrin α5/ITGA5/CD49e Antibody (C-9) is a mouse monoclonal IgG2b κ Integrin α5/ITGA5/CD49e antibody specific for an epitope mapping between amino acids 867-894 at the C-terminus of Integrin α5 of human origin. Species Reactivity: Human, Mouse, Rat.
• Granzyme B (CST, 44153, 1:200): Granzyme B (E5V2L) Rabbit mAb recognizes endogenous levels of total mouse Granzyme B protein. This antibody does not cross-react with human Granzyme B proteins. Non-specific staining was observed in mouse kidney. Monoclonal antibody is produced by immunizing animals with a synthetic peptide corresponding to residues surrounding Ala197 of mouse Granzyme B protein. Species Reactivity: Mouse.
• PD-L1 (CST, 64988, 1:100): PD-L1 (D5V3B) Rabbit mAb recognizes endogenous levels of total mouse PD-L1 protein. Non-specific staining of keratinized epithelium has been observed. Monoclonal antibody is produced by immunizing animals with a synthetic peptide corresponding to residues surrounding Gly216 of mouse PD-L1 protein. Species Reactivity: Mouse.
• Tim3 (CST, 83882, 1:200): TIM-3 (D3M9R) XP® Rabbit mAb recognizes endogenous levels of total TIM-3 protein. Monoclonal antibody is produced by immunizing animals with a synthetic peptide corresponding to residues surrounding Pro137 of mouse TIM-3 protein. Species Reactivity: Mouse.

The following antibodies were used for fluorescent IHC on human tissue:
• PanCK (Ventana, 760-2595, 1:10): Anti-Pan Keratin (AE1/AE3/PCK26) Primary Antibody is an antibody cocktail intended for laboratory use in the qualitative immunohistochemical detection of most acidic cytokeratins and all basic cytokeratins. Species Reactivity: Mouse.
• OPN (Abcam, Ab63856, 1:100): Rabbit polycloncal antibody to Osteopontin IgG isotype; suitable for: ICC/IF, IHC-P, WB. Immunogen: Synthetic peptide corresponding to Human Osteopontin aa 150-250 conjugated to keyhole limpet haemocyanin.Species Reactivity: Mouse, Rat, Human.
• CD68 (Ventana, 790-2931, 1:5): CONFIRM anti-CD68 (KP-1) Primary Antibody is intended for laboratory use in the qualitative immunohistochemical detection of CD68 by light microscopy. Species Reactivity: Mouse.

The following antibodies were used for immunoblots:
• Stat3 (CST, 9139, 1:1000): Stat3 (124H6) Mouse mAb detects endogenous levels of total Stat3 protein. Monoclonal antibody is produced by immunizing animals with a synthetic peptide centered around amino acid Gln692 of human Stat3. Species Reactivity: Human, Mouse, Rat, Monkey.
• p-Stat3 (CST, 9145, 1:1000): Phospho-Stat3 (Tyr705) (D3A7) XP(R) Rabbit monoclonal antibody detects endogenous levels of Stat3 only when phosphorylated at tyrosine 705. This antibody does not cross-react with phospho-EGFR or the corresponding phospho-tyrosines of other Stat proteins. Species Reactivity: Human, Mouse, Rat, Monkey.
• β-actin (Sigma, A5441, 1:2000): Monoclonal Anti-β-Actin antibody produced in mouse, unconjugated, clone AC-15, isotype IgG1. Monoclonal Anti-β-Actin (mouse IgG1 isotype) is derived from the AC-15 hybridoma produced by the fusion of mouse myeloma cells and splenocytes from an immunized mouse. Immunogen: slightly modified β-cytoplasmic actin N-terminal peptide, Ac-Asp-Asp-Asp-Ile-Ala-Ala-Leu-Val-Ile-Asp-Asn-Gly-Ser-Gly-Lys, conjugated to KLH. Species, Reactivity: Sheep, Carp, Feline, Chicken, Rat, Mouse,

Rabbit, Canine, Pig, Human, Bovine, Guinea Pig, Hirudo medicinalis.
• Tubulin (CST, 2148, 1:1000): The α/β-Tubulin Antibody detects endogenous levels of both α- and β-tubulin total protein, and does cross-react with both recombinant α- and β-tubulin. Polyclonal antibodies are produced by immunizing animals with a synthetic peptide corresponding to the sequence of human α- and β-tubulin. Antibodies are purified by protein A and peptide affinity chromatography. Species Reactivity: Human, Mouse, Rat, Monkey, Zebrafish, Bovine.
• β3 integrin (Abcam, ab119992, 1:1000): Recombinant Anti-Integrin beta 3 antibody [EPR2342] produced recombinantly (animal-free) for high batch-to-batch consistency and long term security of supply is suitable for: Flow Cyt (Intra), WB, IP. Immunogen: Synthetic peptide. This information is proprietary to Abcam and/or its suppliers. Species Reactivity: Human, Mouse, Rat.
• αV integrin (CST, 60896, 1:1000): Integrin αV (D2N5H) Rabbit mAb recognizes endogenous levels of total Integrin αV. Monoclonal antibody is produced by immunizing animals with recombinant protein specific to the central region of human integrin αV protein. Species Reactivity: Human, Mouse, Rat, Monkey.
• CD44 (CST, 37259, 1:1000): CD44 (E7K2Y) XP(R) Rabbit mAb recognizes endogenous levels of total CD44 protein. Monoclonal antibody is produced by immunizing animals with a synthetic peptide corresponding to residues surrounding Pro136 of human CD44 protein. This sequence region is conserved in all isoforms of CD44 reported in Uniprot, with the exception of isoform 2 and isoform 19. Species Reactivity: Human, Mouse, Rat.

Fixed cells were stained using the following antibodies for flow cytometry:
• eFluorTM 506 (ThermoFisher/eBioscience 65-0866-14): Fixable Viability Dye eFluor™ 506 is a viability dye that can be used to irreversibly label dead cells prior to cryopreservation, fixation and/or permeabilization procedures. Unlike 7-AAD and propidium iodide, cells labeled with Fixable Viability Dyes can be washed, fixed, permabilized, and stained for intracellular antigens without any loss of staining intensity of the dead cells. Thus, using Fixable Viability Dyes allows dead cells to be excluded from analysis when intracellular targets are being studied. Fixable Viability Dyes may be used to label cells from all species.
• F4/80 (BD bioscience 123114): BioLegend's PE/Cyanine7 anti-mouse F4/80 is a Rat monoclonal antibody. This antibody has been shown to work in applications such as: Flow Cytometry. Species Reactivity: Mouse.
• CD11b (BD bioscience 563168): The M1/70 monoclonal antibody specifically binds to CD11b, also known as Integrin alpha M (Itgam or αM). CD11b is a 170-kDa type 1 transmembrane glycoprotein and belongs to the Integrin alpha chain family. CD11b serves as the alpha chain of the heterodimeric Mac-1 integrin (CD11b/CD18, αMβ2), also known as complement receptor 3 (CR3). Mac-1 mediates adhesion to ICAM-1 (CD54), ICAM-2 (CD102), fibrinogen and binding to C3bi. Mac-1 is expressed at varying levels on granulocytes, macrophages, myeloid-derived dendritic cells, natural killer cells, microglia, and B-1 B lymphocytes. Mac-1 expression is rapidly upregulated on neutrophils after activation, in the same time period that CD62L (L-selectin) is shed from the cell surface. The M1/70 antibody reportedly blocks cell adherence and C3bi binding but does not block cell-mediated lysis. Cross-reaction of the M1/70 antibody with CD11b expressed on human monocytes, polymorphonuclear leukocytes, and NK cells has been reported. The antibody was conjugated to BD Horizon™ BV711 which is part of the BD Horizon Brilliant™ Violet family of dyes. This dye is a tandem fluorochrome of BD Horizon™ BV421 with an Ex Max of 405-nm and an acceptor dye with an Em Max at 711-nm. BD Horizon™ BV711 can be excited by the violet laser and detected in a filter used to detect Cy™5.5 / Alexa Fluor® 700-like dyes (eg, 712/20-nm filter). Due to the excitation and emission characteristics of the acceptor dye, there may be moderate spillover into the Alexa Fluor® 700 and PerCP-Cy™5.5 detectors. However, the spillover can be corrected through compensation as with any other dye combination. Species Reactivity: QC Testing: Mouse, Tested in Development: Human.
• CD206 (Biolegend 141706): PE anti-mouse CD206 (MMR) monoclonal antibody clone C068C2. Immunogen: Recombinant mouse CD206 (MMR). Recommended usage: Each lot of this antibody is quality control tested by intracellular immunofluorescent staining with flow cytometric analysis. For flow cytometric staining, the suggested use of this reagent is ≤0.5 µg per million cells in 100 µl volume. It is recommended that the reagent be titrated for optimal performance for each application. Verified Species Reactivity: Mouse.
• IL-4R (Biolegend 504117): PE/Cyanine7 anti-mouse IL-4 Rat Monoclonal Antibody. The antibody was purified by affinity chromatography and conjugated with PE/Cyanine7 under optimal conditions. Note: BioLegend is in the process of converting the name PE/Cy7 to PE/Cyanine7. The dye molecule remains the same, so you should expect the same quality and performance from our PE/Cyanine7. Species Reactivity: Mouse.
• Arginase 1 (Invitrogen 46-3697-82): The monoclonal antibody A1exF5 recognizes both human and mouse Arginase 1, a cytosolic enzyme (Arg1). This A1exF5 clone is compatible with both, the standard intracellular protocols, and the Foxp3/Transcription Factor Staining Buffer Set. Applications Reported: This A1exF5 antibody has been reported for use in flow cytometric analysis. Species Reactivity: Mouse, Human.

The following antibodies were used for in vivo experiments:
• Recombinant mouse osteopontin (R&D Systems 441-OP): Mouse myeloma cell line, NS0¬derived Leu17¬Asn294 (Glu99Gly), with a C¬terminal 6-His tag Accession # Q547B5. Activity: Measured by the ability of the immobilized protein to support the adhesion of HEK293 human embryonic kidney cells. Agnihotri, R. et al. (2001) J. Biol. Chem. 276:28261. When 1 x 105 cells/well are added to a Recombinant Mouse (rm) Osteopontin/OPN coated plate, cell adhesion is enhanced in a dose-dependent manner after 1-hour incubation at 37 °C. The ED50 for this effect is 0.25-1.5 µg/mL. Recombinant Human Coagulation Factor II/Thrombin (Catalog # 1473-SE) proteolytic treatment of this rmOsteopontin can increase HEK293 cell adhesion by about 10-fold.
• Osteopontin neutralizing antibody (R&D Systems AF808): Mouse Osteopontin/OPN Antibody detects mouse Osteopontin (OPN). Source: Polyclonal Goat IgG, antigen affinity-0purified. Immunogen: Mouse myeloma cell line NS0-derived recombinant mouse Osteopontin/OPN (R&D Systems, Catalog # 441-OP) Leu17-Asn294 (Glu99Gly) Accession # Q547B5. Species Reactivity: Detects mouse OPN in ELISAs and Western Blots.
• Anti-PD-1 (RMP1-14, Bio X cell #BE0146): The RMP1-14 monoclonal antibody reacts with mouse PD-1 (programmed death-1) also known as CD279. PD-1 is a 50-55 kDa cell surface receptor encoded by the Pdcd1 gene that belongs to the CD28 family of the Ig superfamily. PD-1 is transiently expressed on CD4 and CD8 thymocytes as well as activated T and B lymphocytes and myeloid cells. PD-1 expression declines after successful elimination of antigen. Isotype: Rat IgG2aK, Immunogen: Syrian Hamster BKH cells transfected with mouse PD-1 cDNA. Species Reactivity: Mouse.
• Normal Goat IgG Control (R&D Systems AB-108-C): Polyclonal Goat IgG.
• Rat IgG2a (Bio X cell #BE0089): The 2A3 monoclonal antibody reacts with trinitrophenol. Because trinitrophenol is not expressed by mammals this antibody is ideal for use as an isotype-matched control for rat IgG2a antibodies in most in vivo and in vitro applications. Isotype: Rat IgG2aK.

Eukaryotic cell lines

Policy information about cell lines and Sex and Gender in Research

Cell line source(s)	Isolation and culture of mouse bone marrow-derived macrophages (BMDM): Protocol adapted from Helft et al, 2015 [https://pubmed.ncbi.nlm.nih.gov/26084029/]. Virgin female FVB mice were euthanized, and femurs and tibias were collected and kept on ice in 2% heat-inactivated FBS (HI FBS, FBS in 56˚C water bath for 30 min) in PBS. In the tissue culture hood, the epiphyses of femurs were twisted off and the bone marrow was flushed out using a 23G needle and 3 mL syringe with 10% HI FBS BMDM media into a 6 well plate (BMDM media: DMEM with 1X glutamax, 1X sodium pyruvate, 1X β-mercaptoethanol, penicillin (100 U/mL), and streptomycin (100 mg/mL)). Likewise, both ends of tibias were cut off ad the bone marrow flushed out into the same well for the same mouse. Bone marrows were broken by pipetting up and down in 10% HI FBS BMDM media, passed through a 40 μm strainer and centrifuged at 1300 RMP for 5min at 4˚C. All samples were treated with ACK lysis buffer (150 mM NH_4Cl, 10 mM $KHCO_3$, 0.1 mM Na_2EDTA, pH 7.5) to remove red blood cells for 1 min at room temperature. 5 million cells were plated in each 10 cm tissue culture dish in 10% HI FBS BMDM media with 30 ng/mL of M-CSF (Peprotech 315-02). BMDM cells were supplemented with 30 ng/mL of M-CSF on day 3 and day 6. On day 6, BMDM cells were supplemented with saline, 500 ng/mL of rmOPN (R&D Systems 441-OP) and/or 20 ng/mL of IL-4 (Peprotech 214-14) for polarization for 48 hours.<br><br>MMTV-PyV mT wild type and Itgb1 fl/fl cell lines: Mammary tumors from mice of the respective genotype were harvested and dissociated as previously described by Bui et al [https://pubmed.ncbi.nlm.nih.gov/34782719/]. Briefly, Tumors were processed with the McIlwain Tissue Chopper and disassociated in DMEM with 2.4 mg/mL Collagenase B (Roche) and 2.4 mg/mL Dispase II (Roche) for 2 hours rotating at 37°C. Dissociated cells were washed with ACK lysis buffer (150 mM NH_4Cl, 10 mM $KHCO_3$, 0.1 mM Na_2EDTA, pH 7.5) to remove red blood cells, then in 1 mM EDTA in PBS, passed through cell strainer, and plated on plastic. Cells were maintained in complete media (DMEM with EGF (5 ng/mL), bovine pituitary extract (35 μg/mL), insulin (5 μg/mL), hydrocortisone (1 μg/mL), penicillin (100 units/mL), streptomycin (100 μg/mL), gentamicin (50 μg/mL)) supplemented with 5% vol/vol FBS.
Authentication	BMDMs: F4/80 expression was validated by flow cytometry.<br>MMTV-PyV mT wild type and Itgb1 fl/fl cells lines: genotypes were validated by genomic DNA PCR.<br>More details can be found in the manuscript.
Mycoplasma contamination	All cells used in this study were negative for mycoplasma contamination tested using the MycoAlert Kit (Lonza, LT07-118).
Commonly misidentified lines<br>(See ICLAC register)	This study did not use any commonly misidentified lines.

Animals and other research organisms

Policy information about studies involving animals; ARRIVE guidelines recommended for reporting animal research, and Sex and Gender in Research

Laboratory animals	MIC and MMTV-PyV mT transgenic mice, including Itgb1 (β1 integrin) floxed alleles and Stat3 floxed alleles were bred and maintained on a pure FVB/N background. Genomic DNA was extracted from tails of all mice using crude salt extraction and subsequently used for genotype confirmation using PCR. Experimental and control animals were given drinking water with doxycycline (2 mg/mL) at 9 to 12 weeks of age ("induction"), weighed and monitored weekly by physical palpations for tumor formation. All mice from animal experiments in this study were housed and handled at the Comparative Medicine and Animal Resource Centre at McGill University, approved by and in compliance with the Animal Ethics Committee, Facility Animal Care Committee, and Canadian Council on Animal Care (Animal Use Protocol #MCGL5518). All mice were housed at a maximum of five animals per cages with fluid and food ad libitum, on a 12 hour dark/light cycle, at ambient temperature, and a relative humidity of 45% to 65%. All mice were euthanized prior to or at the approved tumor volume endpoint of 2.5 cm^3 for a single tumor mass or a total of 5 cm^3 for multifocal tumors. Only female mice were used experimentally as this study pertains to female breast cancer.
Wild animals	This study did not use any wild animals.
Reporting on sex	This study takes into consideration that breast cancer predominantly affects women although we acknowledge that 1% of cases occur in men. Therefore, our study solely uses female experimental mice to recapitulate breast cancer in women. All experimental mice were females, on the FVB/N background. No data was collected on gender.
Field-collected samples	This study did not use any filed-collected samples.
Ethics oversight	Experiments involving mice were conducted in accordance with McGill University and Canadian Council on Animal Care (CCAC) ethical guidelines under a protocol (MCGL-5518) approved by the McGill University Downtown Campus Facility Animal Care Committee (FACC), a branch of the McGill University Animal Care Committee (UACC), Montreal, QC, Canada.

Note that full information on the approval of the study protocol must also be provided in the manuscript.

Plants

Seed stocks	No plant specimens were used in this study.
Novel plant genotypes	No plant specimens were used in this study.
Authentication	No plant specimens were used in this study.

Flow Cytometry

Plots

Confirm that:

- [ ] The axis labels state the marker and fluorochrome used (e.g. CD4-FITC).
- [x] The axis scales are clearly visible. Include numbers along axes only for bottom left plot of group (a 'group' is an analysis of identical markers).
- [x] All plots are contour plots with outliers or pseudocolor plots.
- [x] A numerical value for number of cells or percentage (with statistics) is provided.

Methodology

Sample preparation	Mouse BMDM cells were extracted and cultured as previously described in "Eukaryotic cell lines". BMDMs were fixed using BD Cytofix/CytopermTM Plus (BD Bioscience, #555028) according to manufacturer's protocol, Fixed cells were stained for viability using the fixable viability dye eFluorTM 506 (ThermoFisher/eBioscience 65-0866-14), F4/80 (BD bioscience 123114), CD11b (Biolegend 563168), CD206 (Biolegend 141706), IL-4R (Biolegend 504117) and Arginase 1 (Invitrogen 46-3697-82).
Instrument	BD LSR Fortessa flow cytometer was used for data collection.
Software	Flowjo 10.6.2 was used for data analysis.
Cell population abundance	All samples had at least 51%-80% F4/80+ CD11b+ macrophages out of all live cells.
Gating strategy	Cells were gated as follow: Total F4/80+ CD11b+ macrophages were gated out of all live cells, and sorted based on the expression of IL-4 receptor (IL-4R) and arginase 1.

- [ ] Tick this box to confirm that a figure exemplifying the gating strategy is provided in the Supplementary Information.
